# N–O Cleavage reactions of heterobicycloalkene-fused 2-isoxazolines

**DOI:** 10.3762/bjoc.10.227

**Published:** 2014-09-16

**Authors:** Jaipal R Nagireddy, Geoffrey K Tranmer, Emily Carlson, William Tam

**Affiliations:** 1 Guelph-Waterloo Centre for Graduate Work in Chemistry and Biochemistry, Department of Chemistry and Biochemistry, University of Guelph, Guelph, Ontario, N1G 2W1, Canada

**Keywords:** azabicycloalkene, β-hydroxyketone, 2-isoxazoline, oxabicycloalkene, Raney nickel

## Abstract

Transition metal-mediated N–O bond cleavage reactions of heterobicycloalkene-fused 3-methyl-2-isoxazolines were investigated. Optimal cleavage conditions were found with Raney nickel/AlCl_3_ mediation in aqueous methanol. The reaction provided a diverse collection of novel heterobicycle-fused β-hydroxyketones with good to excellent yields (66–95%) and without the need for chromatographic purification.

## Introduction

**Scheme 1:**
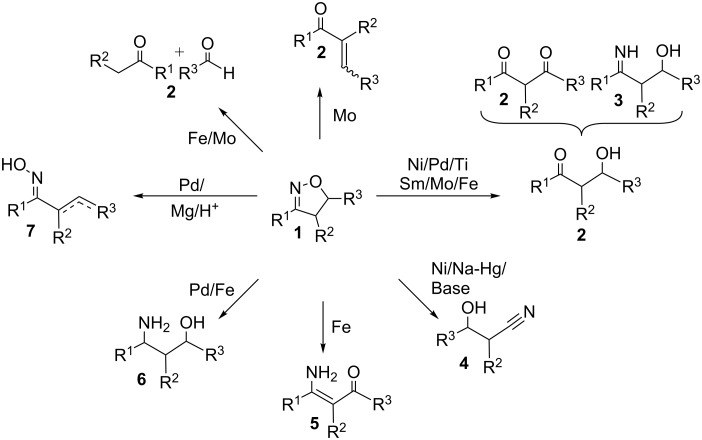
Cleavage reactions of 2-isoxazoline **1**.

2-Isoxazolines **1** are practical precursors to countless structural motifs found in nature. Various carbonyl compounds **2**, β-hydroxyimines **3**, β-hydroxynitriles **4**, β-aminoketones **5**, γ-aminoalcohols **6** and oximes **7** can all be readily attained through ring cleavage of 2-isoxazolines using suitable reagents for each transformation (Scheme 1) [1–7]. In recent years, there has been considerable interest in applying this isoxazoline-cleavage methodology to the preparation of biologically active natural products, including novel antibacterial, antifungal and anticancer treatments [8–11]. Some common methods of reductive N–O bond cleavage in isoxazolines include hydrogenolysis using Raney nickel, reduction by LiAlH_4_, TiCl_3_, SmI_2_, or treatment with Mo(CO)_6_ [12–19]. Although cleavage reactions of 2-isoxazolines have been extensively studied, little is still known regarding cleavage reactions of 2-isoxazolines fused to bicyclic compounds.

We have previously shown that the Mo(CO)_6_-mediated cleavage of carbobicycle-fused 2-isoxazolines converts both *exo*- and *endo* stereoisomers of **8** into the corresponding single isomer of cyclopentene **9** (Table 1) [20].

**Table 1:** Molybdenum-mediated cleavage of carbobicycle-fused isoxazolines.

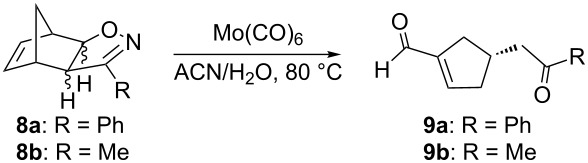				

Entry	R	Isoxazoline	Product	Yield (%)^a^

1	Ph	*exo*-**8a**	**9a**	66
2	Ph	*endo***-8a**	**9a**	70
3	Me	*exo*-**8b**	**9b**	61
4	Me	*endo-***8b**	**9b**	62

^a^Isolated yield after column chromatography.

**Scheme 2:**
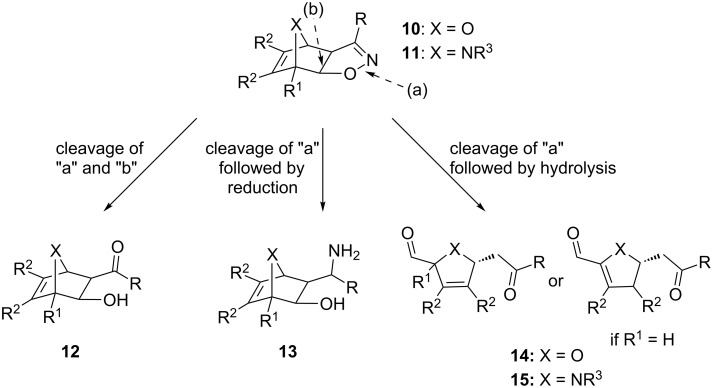
Potential modes of cleavage for heterobicycloalkene-fused isoxazolines **10** or **11**.

Since then, we have been interested in the outcomes of cleavage reactions of 2-isoxazolines fused to heterobicyclic compounds **10** or **11**, which we were able to prepare in a highly regio- and stereoselective manner [21]. Selective cleavage of the N–O bond or simultaneous cleavage of both N–O and bridge C–C bonds of these isoxazolines could provide novel routes to the aforementioned functionalities attached to heterobicyclic ring systems such as β-hydroxycarbonyl derivatives **12** and γ-aminoalcohols **13**, as well as carbonyl compounds including phthalan **14** and isoindoline derivatives **15** (R^2^ = Ar; Scheme 2).

## Results and Discussion

For our investigation of heterobicycloalkene-fused 2-isoxazoline cleavage, we initially planned to follow our former Mo(CO)_6_-promoted approach. We elected substrate **10a** (Scheme 2, R’ = Me, R = X = Y = H) for early optimization trials. The molybdenum-mediated conditions, however, were unsuccessful at both the previously optimized temperature of 80 °C (which led to extensive degradation) and at lower temperatures of 45–60 °C (where only **10a** was recovered). We then adopted the methodology of Simoni and coworkers, treating **10a** with refluxing Mo(CO)_6_ in acetonitrile [18]. Unfortunately, in our studies this showed no noticeable improvement, as the reaction did not proceed at room temperature, and at 45–60 °C we observed a series of spots by TLC which were not separable by column chromatography. This prompted us to seek other reagents capable of inducing N–O cleavage. We subsequently attempted hydrogenation using Pd/C with various solvent systems (MeOH, MeOH/AcOH, AcOH/H_2_O) as well as hydrogen sources (H_2_, ammonium formate) [22], as well as the combination of Pd(OH)_2_ with H_2_. However, none of these palladium-mediated attempts resulted in a clean transformation. Trials involving zinc dust in aqueous AcOH, as well as iron and ammonium chloride in aqueous EtOH at various temperatures also failed [23–24]. Finally, we turned to nickel-mediated reactions.

As before, our early trials with Raney nickel/H_2_ in MeOH and Raney nickel in AcOH showed no signs of progress. However, under conditions of Raney nickel/AlCl_3_ in a 5:1 MeOH/H_2_O mixture [25–26], we observed a clean transformation and the product readily precipitated out of solution following work-up, allowing us to bypass chromatographic purification. The product so obtained was found to be β-hydroxyketone **16a** (Table 2, entry 1).

Once the optimal conditions were found, various symmetrical heterobicycle-fused 2-isoxazolines were opened by Raney-Ni/AlCl_3_ mediation. These results are summarized in Table 2. Compared to the parent compound **10a** which gave an appreciable 93% yield of the corresponding β-hydroxyketone (Table 2, entry 1), replacement of two of the arene hydrogens for bromine atoms in **10b** resulted in a lower tendency toward N–O bond cleavage (Table 2, entry 2). In addition, while both 6,7*-* and 5,8*-*dimethoxy-substituted compounds **10c** and **10d** gave excellent yields of 87% and 94%, respectively (Table 2, entries 4 and 5), the 5,8*-*dimethyl derivative **10e** was slightly lower yielding, providing 76% isolated product (Table 2, entry 6). Overall, isoxazolines **10a**–**e** all showed good conversion to their β-hydroxyketones. We then proceeded to expand the reaction scope to include unsymmetrical substrates, namely those with bridgehead (C1) substituents.

**Table 2:** Effects of arene substitution on Raney Ni/AlCl_3_-mediated cleavage of symmetrical 7-oxabenzonorbornadiene-fused 3-methyl-2-isoxazolines.

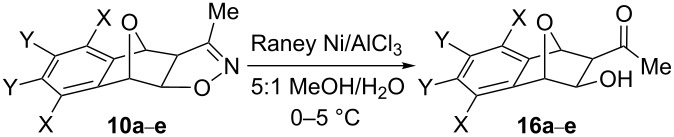					

Entry	X	Y	Isoxazoline	Product	Yield (%)^a^

1	H	H	**10a**	**16a**	93
2	H	Br	**10b**	**16b**	71
3	H	OMe	**10c**	**16c**	87
4	OMe	H	**10d**	**16d**	94
5	Me	H	**10e**	**16e**	76

^a^Isolated yield.

The results of isoxazoline cleavage on unsymmetrical oxabicyclic alkene-fused systems are summarized in Table 3. Due to decreased solubility in aqueous methanol, the C1-substituted compounds **10f–k** were reacted in a 5:5:2 THF/MeOH/H_2_O solvent system. Relative to the unsubstituted parent **10a** (Table 3, entry 1), reactions of isoxazolines **10f** and **10g** bearing alkyl substituents in the C1 position were still reasonably good (Table 3, entries 2 and 3), and even with electron-withdrawing C1 substituents in compounds **10h–j**, the yields did not suffer to any extreme (Table 3, entries 4–6). In addition, the transformation to β-hydroxyketone succeeded with compound **10k** bearing both arene and C1 substituents to furnish **16k** (Table 3, entry 7) whose structure was confirmed by X-ray crystallographic analysis [27].

**Table 3:** Effects of bridgehead substitution on Raney Ni/AlCl_3_ mediated cleavage of 7-oxabenzonorbornadiene-fused 3-methyl-2-isoxazolines.

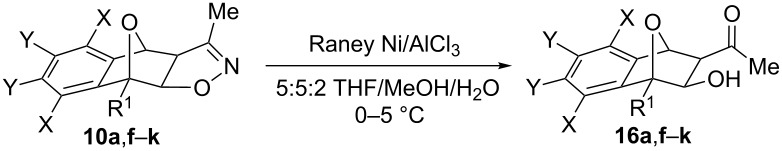						

Entry	R^1^	X	Y	Isoxazoline	Product	Yield (%)^a^

1^b^	H	H	H	**10a**	**16a**	93
2	Me	H	H	**10f**	**16f**	80
3	Et	H	H	**10g**	**16g**	95
4	CH_2_OH	H	H	**10h**	**16h**	85
5	C(O)CH_3_	H	H	**10i**	**16i**	69
6	COOMe	H	H	**10j**	**16j**	85
7	Me	OMe	H	**10k**	**16k**	86

^a^Isolated yield. ^b^5:1 MeOH/H_2_O solvent system.

**Scheme 3:**
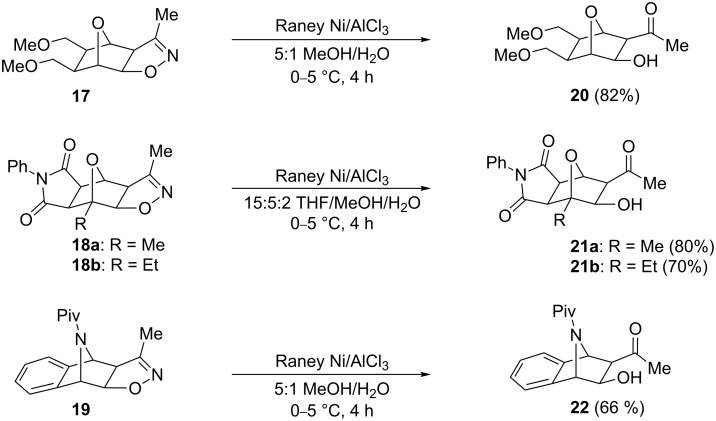
Raney Ni/AlCl_3_-mediated cleavage of 2-isoxazolines with various fused heterobicyclic frameworks.

Finally, we applied this N–O bond-cleavage methodology to several other heterobicycle-fused 2-isoxazolines to verify its compatibility with similar structures (Scheme 3). As with isoxazolines **10**, each of these compounds (**17**, **18** and **19**) was prepared in our laboratories by a combination of Diels–Alder cycloaddition and 1,3-dipolar cycloaddition reactions [21]. We found that the Raney nickel-mediated cleavage took place readily on **17** to yield a β-hydroxyketo-functionalized 7-oxanorbornane **20** (Scheme 3), and was also successful with isoxazolines **18a** and **18b**, affording *N*-phenylmaleimide-fused **21a** and **21b** (where the solvent ratio was modified to account for the lower solubility of **18a**/**18b** in aqueous methanol). Furthermore, the reaction was also successful in the conversion of 7-azanorbornadiene-fused **19** to **22**, suggesting the potential utility of the isoxazoline-cleavage reaction with other azabicyclic systems.

**Scheme 4:**
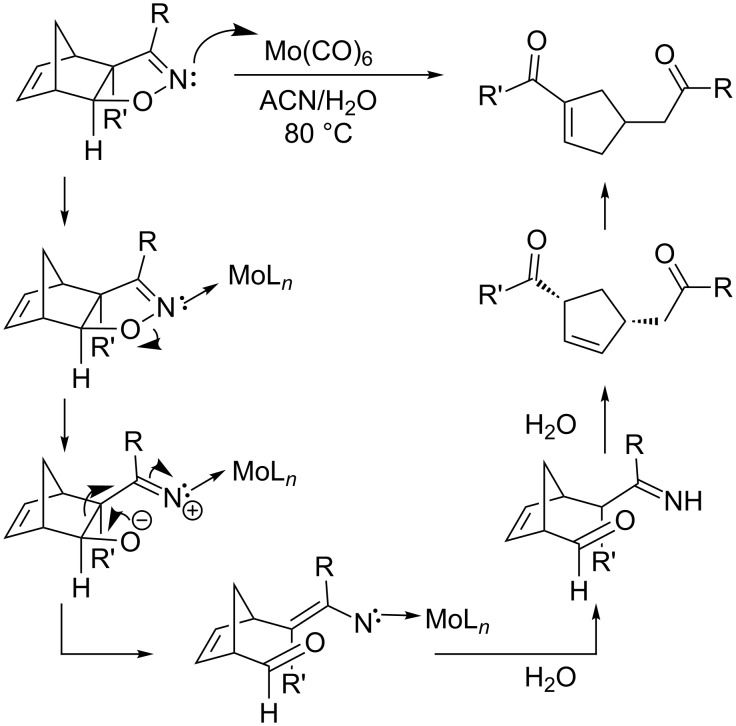
Proposed mechanism for Mo-mediated cleavage of carbobicycle-fused 2-isoxazoline.

In our initial report of molybdenum-mediated cleavage of carbobicycloalkene-fused isoxazolines **8**, we proposed a general mechanism of the transformation to attached ring system **9** (Scheme 4). As the carbobicyclic framework contains no metal-coordinatable atom, we assumed that the cleavage reaction proceeds with initial coordination of the nitrogen atom to molybdenum, as suggeseted by Simoni and coworkers [18].

**Scheme 5:**
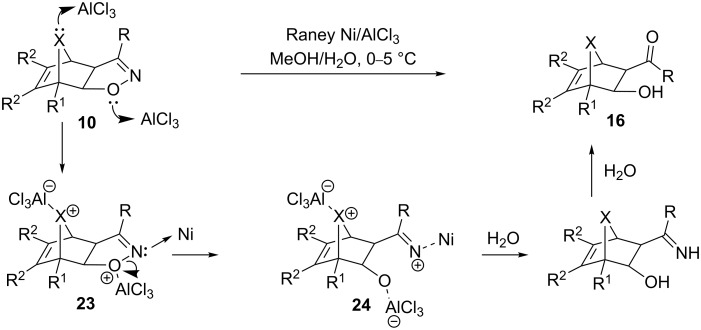
Proposed mechanism for Raney nickel-mediated formation of β-hydroxyketones from heterobicycloalkene-fused 2-isoxazolines.

In comparison, the β-hydroxyketones **16** generated in the current investigation suggest both reductive N–O cleavage and imine hydrolysis occurring in a single pot. Moreover, there is no indication of a retro-aldol cleavage. Since molybdenum catalysis was ineffective with the heterobicycloalkene-fused isoxazolines, it is possible that molybdenum preferentially coordinates to the heteroatom of the bicycloalkene rather than to the nitrogen of the isoxazoline, reducing its tendency toward N–O cleavage. Furthermore, since the reaction did not proceed in the absence of either AlCl_3_ or nickel, coordination of both metals appears to be an essential step in the formation of **16**. Under the optimized conditions, we suggest that initial binding of Lewis acidic aluminum to the bridge heteroatom of **10** leaves its isoxazoline nitrogen free to interact with the Raney nickel (Scheme 5, **23**). The N–O bond is subsequently broken to afford **24**, and it is conceivable at this stage that the added coordination of AlCl_3_ to the alkoxy oxygen of **24** prevents retro-aldol cleavage from taking place. Finally, in a manner analogous to our former proposal, hydrolysis ensues to produce **16** (Scheme 4).

## Conclusion

To conclude, we have demonstrated the first examples of Raney nickel/AlCl_3_-mediated cleavage reactions of 2-isoxazoline rings fused to oxabicyclic and azabicyclic frameworks, which show novel reactivity compared to our former report of carbobicyclic analogues. The transformation involves N–O bond cleavage of the 2-isoxazoline followed by imine hydrolysis, leading to β-hydroxyketone products with moderate to excellent yields. We have investigated the efficacy of various cleavage conditions and found that although none of Mo(CO)_6_, Zn/AcOH, Fe/NH_4_Cl, Pd-C/H_2_, Pd(OH)_2_/H_2_ or Raney nickel/AcOH/H_2_ is suitable for cleavage of 2-isoxazolines fused to heterobicyclic frameworks, the combination of Raney nickel/AlCl_3_ is a suitable mediator for N–O bond cleavage in a variety of heterobicycloalkene-fused 2-isoxazoline systems.

## Supporting Information

Experimental procedures and copies of ^1^H and ^13^C NMR spectra for compounds **16a–k**, **20**, **21a**,**b** and **22**.

File 1 Experimental.

File 2 NMR Spectra.
