# Supplementary material for: N–O Cleavage reactions of heterobicycloalkene-fused 2-isoxazolines
Source: Beilstein J Org Chem. 2014 Sep 16;10:2200–5. doi: 10.3762/bjoc.10.227 (PMC4168885; doi:10.3762/bjoc.10.227)
Supplement: File 1 — Experimental. [file Beilstein_J_Org_Chem-10-2200-s001.pdf]

Supporting Information

for

N–O Cleavage reactions of heterobicycloalkene-fused 2-isoxazolines

Jaipal R. Nagireddy, Geoffrey K. Tranmer, Emily Carlson and William Tam*

Address: Guelph-Waterloo Centre for Graduate Work in Chemistry and Biochemistry, Department of Chemistry and Biochemistry, University of Guelph, Guelph, Ontario, N1G 2W1, Canada

Email: William Tam - wtam@uoguelph.ca

*Corresponding author

Experimental

Table of Contents

General considerations **S2**

Previously reported key compounds **8**, **10**, **11** and **9a-b** **S2**

General procedure for cleavage of symmetrical 2-isoxazolines **10a-e**; **17** and **19** **S3**

General procedure for cleavage of unsymmetrical 2-isoxazolines **10f-k**; **18a-b** **S3**

Characterization data for compounds **16a-k** **S4-S7**

Characterization data for compounds **20**, **21a-b**, **22** **S7-S8**

General considerations

All reactions were performed in septum-sealed, flame-dried flasks under nitrogen. All commercial reagents were used as received from their respective suppliers. Column chromatography was performed on 230-400 mesh silica gel using flash column chromatography techniques.[1] Analytical thin-layer chromatography (TLC) was performed on Merck pre-coated silica gel 60 F_{254} plates. Infrared samples were prepared as thin films on KBr disks and spectra were recorded on a Bomem MB-100 FTIR spectrophotometer. ^{1}H and ^{13}C NMR spectra were recorded on Bruker Avance 300 or 400 MHz spectrometers. Chemical shift (δ) values are reported in parts per million (ppm) from tetramethylsilane with the solvent resonance as the internal standard (deuterochloroform, ^{1}H: δ 7.24 ppm; ^{13}C: 77.0 ppm). HRMS analyses were performed at the Queen's Mass Spectrometry and Proteomics Unit, Kingston, Ontario.

Experimental Procedures and Full Characterization Data for Previously Reported Key Compounds. For complete experimental procedures and full characterization data of all bicycloalkene-fused 2-isoxazolines **8**, **10**, **11** and cyclopentenes **9a-b**, see the Supplementary Material section of our previous reports on the 1,3-dipolar cycloaddition of nitrile oxides with norbornadiene,[2] and 7-oxa- and 7-azabenzonorbornadienes.[3]

---

[1] Still, W.C.; Kahn, M.; Mitra, A. *J. Org. Chem.* **1978,** *43,* 2923-2925. doi:10.1021/jo00408a041
[2] Tam, W.; Tranmer, G.K. *Org. Lett.* **2002,** *4,* 4101-4104. doi: 10.1021/ol026846k
[3] Nagireddy, J.R.; Carlson, E.; Tam, W. *Can. J. Chem.* **2014,** *97,* 635-639. doi: 10.1139/cjc-2014-0140

General Procedures

Cleavage of Symmetrical 2-isoxazolines 10a-e (Table 2, entries 1-5); 17 and 19 (Scheme 4). In a similar manner to our recent publication,[4] methanol (25.0 mL) and distilled water (5.0 mL) was added to an oven-dried flask containing the isoxazoline (102 mg, 0.5 mmol), and the mixture was cooled to 0-5 ˚C. $AlCl_3$ (200 mg, 1.5 mmol) was added to the cold solution in one portion and maintained for 15 minutes. Raney-nickel (1.2 g) was added and the reaction was stirred for 4 hours. The solution was filtered through Celite, the filter cake was washed with methanol, and the solvent was then removed by rotary evaporation. The organic mixture was extracted with dichloromethane, dried over sodium sulphate and reconcentrated. After addition of hexanes (3-5 mL), the crude product was allowed to stir at room temperature for 30 minutes and was then filtered to afford pure β-hydroxyketone (95 mg, 0.465 mmol, 93%) as a white solid.

Cleavage of Unsymmetrical 2-isoxazolines 10f-k (Table 3, entries 2-7); 18a-b (Scheme 4). Following the above General Procedure, THF was added as a co-solvent of varying quantities: For every 0.15 mmol of isoxazoline **10f-k**, the relative solvent ratio was modified to 5:5:2 (THF:MeOH:water) using THF (5 mL), MeOH (5 mL), and distilled water (2 mL). For every 0.15 mmol of isoxazolines **18a-b**, the solvent ratio was modified to 15:5:2 with THF (15 mL), MeOH (5 mL) and distilled water (2 mL).

[4] Lough, A.J.; Nagireddy, J.R.; Tam, W. *Acta Cryst.* **2014,** *E70*, o545-o545. doi:10.1107/S1600536814007752

1-(3-Hydroxy-1,2,3,4-tetrahydro-1,4-epoxynaphthalen-2-yl)ethan-1-one, 16a (Table 2, entry 1): Yield = 93% (95 mg, 0.465 mmol); Off-white solid; mp: 137-140 ˚C; R_f = 0.2 (EtOAc-hexanes, 4:6); IR (ν, cm^{-1}): 3055, 2987, 1712, 1442, 1265, 738; ^{1}H NMR (400MHz, $CDCl_3$): δ 7.32-7.30 (m, 1H), 7.18-7.14 (m, 3H), 5.54 (s, 1H), 5.17 (s, 1H); 4.39 (dd, *J* = 10.8 Hz, 7.0 Hz, 1H), 2.95 (d, *J* = 6.8 Hz, 1H), 2.72 (d, *J* = 10.8 Hz, 1H), 2.25 (s, 3H); ^{13}C NMR (100 MHz, $CDCl_3$): δ 207.3, 145.7, 141.6, 127.8, 127.1, 121.1, 118.9, 85.8, 79.7, 73.8, 58.1, 31.6; HRMS (ESI) calcd. for $C_{12}H_{11}O_3$ $[M-H]^-$: 203.0708; found: 303.0701.

1-(6,7-Dibromo-3-hydroxy-1,2,3,4-tetrahydro-1,4-epoxynaphthalen-2-yl)ethan-1-one, 16b (Table 2, entry 2): Yield = 71% (30 mg, 0.082 mmol); Pale yellow solid; mp: 162-164 ˚C; R_f = 0.22 (EtOAc-hexanes, 4:6); IR (ν, cm^{-1}): 3054, 2986, 1715, 1422, 1265, 1026, 896, 739; ^{1}H NMR (400MHz, $CDCl_3$): δ 7.57 (s, 1H), 7.47 (s, 1H), 5.51 (s, 1H), 5.12 (s, 1H), 4.39 (dd, *J* = 10.9 Hz, 6.6 Hz, 1H), 2.96 (d, *J* = 6.5 Hz, 1H), 2.46 (d, *J* = 10.9 Hz, 1H), 2.25 (s, 3H); ^{13}C NMR (100 MHz, $CDCl_3$): δ 206.3, 146.7, 142.6, 126.4, 124.4, 123.9, 123.1, 85.1, 79.2, 73.3, 57.6, 31.5; HRMS (ESI) calcd. for $C_{12}H_9Br_2O_3$ $[M-H]^-$: 358.8918; found: 358.8911.

1-(-3-Hydroxy-6,7-dimethoxy-1,2,3,4-tetrahydro-1,4-epoxynaphthalen-2-yl)ethan-1-one, 16c (Table 2, entry 3): Yield = 87% (60 mg, 0.227 mmol); Off-white solid; mp: 144-145 ˚C; R_f = 0.12 (EtOAc-hexanes, 6:4); IR (ν, cm^{-1}): 3055, 2987, 1712, 1492, 1266, 1090, 739; ^{1}H NMR (400MHz, $CDCl_3$): δ 6.90 (s, 1H), 6.80 (s, 1H), 5.47 (s, 1H), 5.10 (s, 1H), 4.33(m, 1H), 3.85 (s, 3H), 3.84 (s, 3H), 2.90 (d, *J* = 6.7 Hz, 1H), 2.61 (br s, 1H), 2.24 (s, 3H); ^{13}C NMR (100 MHz, $CDCl_3$): δ 207.5, 148.8, 148.2, 138.1, 133.7, 105.5, 103.5, 86.0, 80.0, 74.1, 58.5, 56.4, 56.3, 31.5; HRMS (ESI) calcd. for $C_{14}H_{16}O_5$ $[M-H]^-$: 263.0925; found: 263.0928

1-(3-Hydroxy-5,8-dimethoxy-1,2,3,4-tetrahydro-1,4-epoxynaphthalen-2-yl)ethan-1-one, 16d (Table 2, entry 4): Yield = 94% (38 mg, 0.143); Off-white solid; mp: 126-128 ˚C; R_f = 0.2 (EtOAc-hexanes, 4:6); IR (ν, cm^{-1}): 3054, 2987, 1716, 1501, 1265, 739; ^{1}H NMR (400MHz, $CDCl_3$): δ 6.66-6.61 (m, 2H), 5.66 (s, 1H), 5.31 (s, 1H), 4.39 (dd, *J* = 10.6 Hz, 6.9 Hz, 1H), 3.77 (s, 3H), 3.75 (s, 3H), 2.96 (d, *J* = 6.7 Hz, 1H), 2.56 (d, *J* = 10.7 Hz, 1H), 2.25 (s, 3H); ^{13}C NMR (100 MHz, $CDCl_3$): δ 207.5, 147.9, 146.2, 134.8, 130.5, 112.1, 111.4, 83.8, 77.8, 73.5, 57.3, 56.0, 31.5; HRMS (EI) calcd. for $C_{13}H_{16}O_5$ $[M-H]^-$: 263.0919; found: 263.0913.

1-(3-Hydroxy-5,8-dimethyl-1,2,3,4-tetrahydro-1,4-epoxynaphthalen-2-yl)ethan-1-one, 16e (Table 2, entry 5): Yield = 76% (35 mg, 0.15 mmol); White solid; mp: 155-157 ˚C; R_f = 0.25 (EtOAc-hexanes, 4:6); IR (ν, cm^{-1}): 3054, 2987, 1713, 1422, 1265, 896, 740; ^{1}H NMR (400MHz, $CDCl_3$): δ 6.88-6.83 (m, 2H), 5.58 (s, 1H), 5.21 (s, 1H), 4.36 (dd, *J* = 11.1 Hz, 6.6 Hz, 1H), 2.92 (d, *J* = 6.8 Hz, 1H), 2.70 (d, *J* = 11.1 Hz, 1H), 2.29 (s, 3H), 2.26 (s, 3H), 2.25 (s, 3H); ^{13}C NMR ($CDCl_3$, 100 MHz): δ 207.5, 143.9, 139.7, 129.0, 128.4, 128.3, 126.0, 84.8, 78.5, 73.4, 57.4, 31.5, 18.0, 17.8; HRMS (ESI) calcd. for $C_{14}H_{15}O_3$ $[M-H]^-$: 231.1021; found: 231.1030.

1-(3-Hydroxy-4-methyl-1,2,3,4-tetrahydro-1,4-epoxynaphthalen-2-yl)ethan-1-one, 16f (Table 3, entry 2): Yield = 80% (25 mg, 0.114 mmol); White solid; mp: 161-163 ˚C; R_f = 0.12 (EtOAc-hexanes, 3:7) IR (ν, cm^{-1}): 3420, 3055, 2987, 1710, 1422, 1265, 896, 738; ^{1}H NMR (400MHz, $CDCl_3$): δ 7.22-7.14 (m, 4H), 5.42 (s, 1H), 4.13 (dd, *J* = 10.7 Hz, 6.9 Hz, 1H), 3.05 (d, *J* = 6.9 Hz, 1H), 2.50 (d, *J* = 10.9 Hz, 1H), 2.26 (s, 3H), 1.76 (s, 3H); ^{13}C NMR (100 MHz, $CDCl_3$): δ 207.8, 146.6, 145.0, 127.3, 127.1, 120.0, 118.5, 89.1, 78.7, 75.2, 59.2, 31.6, 12.9; HRMS (ESI) calcd. for $C_{13}H_{15}O_3$ $[M+H]^+$: 219.1016; found: 219.1021.

1-(4-Ethyl-3-hydroxy-1,2,3,4-tetrahydro-1,4-epoxynaphthalen-2-yl)ethan-1-one, 16g (Table 3, entry 3): Yield = 95% (38 mg, 0.163 mmol); White solid; mp: 171-173 ˚C; R_f = 0.3 (EtOAc-hexanes, 3:7); IR (ν, cm^{-1}): 3419, 3054, 2987, 1701, 1635, 1422, 1265, 896, 739; ^{1}H NMR (400MHz, $CDCl_3$): δ 7.25-7.18 (m, 4H), 5.43 (s, 1H), 4.17 (dd, *J* = 11.1 Hz, 6.8 Hz, 1H), 3.03 (d, *J* = 6.8 Hz, 1H), 2.44 (d, *J* = 11.2 Hz, 1H), 2.33-2.24 (m, 1H), 2.25 (s, 3H), 2.19-2.09 (m, 1H), 1.09 (t, *J* = 7.6 Hz, 3H); ^{13}C NMR (100 MHz, $CDCl_3$): δ 207.9, 147.2, 143.3, 127.5, 127.0, 120.8, 118.7, 92.7, 78.6, 74.9, 59.3, 31.6, 19.8, 8.72; HRMS (ESI) calcd. for $C_{14}H_{17}O_3$ $[M+H]^+$: 233.1172; found: 233.1168.

1-(-3-Hydroxy-4-(hydroxymethyl)-1,2,3,4-tetrahydro-1,4-epoxynaphthalen-2-yl)ethan-1-one, 16h (Table 3, entry 4): Yield = 85% (35 mg, 0.15 mmol); White solid; mp: 160-163 ˚C; R_f = 0.12 (EtOAc-hexanes, 1:1); IR (ν, cm^{-1}): 3054, 2987, 1705, 1422, 1265, 896, 739; ^{1}H NMR ($CDCl_3$, 400MHz): 7.34-7.32 (m, 1H), 7.20-7.18 (m, 3H), 5.53 (s, 1H), 4.50-4.40 (m, 3H), 3.04 (d, *J* = 6.9 Hz, 1H), 2.27 (s, 3H); ^{13}C NMR (100 MHz, DMSO-d_6): δ 205.8, 147.3, 144.5, 126.7, 126.1, 121.0, 118.7, 90.9, 77.5, 73.0, 59.0, 58.1, 30.4; HRMS (ESI) calcd. for $C_{13}H_{15}O_4$ $[M+H]^+$: 235.0970; found: 235.0972.

1,1'-(-2-Hydroxy-3,4-dihydro-1,4-epoxynaphthalene-1,3(2*H*)-diyl)bis(ethan-1-one), 16i (Table 3, entry 5): Yield = 69% (9 mg, 0.036 mmol); White solid; mp: 105-110 ˚C; R_f = 0.29 (EtOAc-hexanes, 3:7, 2 elutions); IR (ν, cm^{-1}): 3054, 2987, 1715, 1635, 1422, 1265, 896, 742; ^{1}H NMR (400MHz, $CDCl_3$): δ 7.34 (d, *J* = 7.2 Hz, 1H), 7.23-7.15 (m, 3H), 5.62 (s, 1H), 4.60 (dd, *J* = 7.2 Hz, 6.8 Hz, 1H), 2.98-2.95 (m, 2H), 2.38 (s, 3H), 2.27 (s, 3H); ^{13}C NMR (100 MHz, $CDCl_3$): δ 206.7, 206.0, 145.0, 140.7, 128.4, 127.3, 120.5, 119.1, 96.0, 79.1, 75.4, 58.0, 31.3, 28.8; HRMS (EI) calcd. for $C_{14}H_{14}O_4$ (M+): 246.0892; found: 246.0887.

Methyl 3-acetyl-2-hydroxy-3,4-dihydro-1,4-epoxynaphthalene-1(2*H*)-carboxylate, 16j (Table 3, entry 6): Yield = 85% (25 mg, 0.095 mmol); White solid; mp: 92-95 ˚C; R_f = 0.29 (EtOAc-hexanes, 1:1); IR (ν, cm^{-1}): 3054, 2987, 1763, 1716, 1442, 1265, 896, 735; ^{1}H NMR (400MHz, $CDCl_3$): δ 7.51 (d, *J* = 6.4 Hz, 1H), 7.25-7.19 (m, 3H), 5.60 (s, 1H), 4.56 (dd, *J* = 10.0 Hz, 6.8 Hz, 1H), 3.95 (s, 3H), 3.0 (d, *J* = 7.2 Hz, 1H), 2.95 (d, *J* = 10.0 Hz, 1H), 2.27 (s, 3H); ^{13}C NMR (100 MHz, $CDCl_3$): δ 206.2, 167.2, 144.8, 139.9, 128.6, 127.3, 121.2, 119.0, 91.8, 79.2, 75.5, 57.9, 52.8, 31.4; HRMS (ESI) calcd. for $C_{14}H_{15}O_5$ $[M+H]^+$: 263.0919; found: 263.0915.

1-(3-Hydroxy-5,8-dimethoxy-4-methyl-1,2,3,4-tetrahydro-1,4-epoxynaphthalen-2-yl)ethan-1-one, 16k (Table 3, entry 7): Yield = 86% (52 mg, 0.187 mmol); White solid; mp: 130-132 ˚C; R_f = 0.24 (EtOAc-hexanes, 3:7); IR (ν, cm^{-1}): 3055, 2987, 1712, 1500, 1442, 1265, 896, 736; ^{1}H NMR (400MHz, $CDCl_3$): δ 6.64 (s, 2H), 5.51 (s, 1H), 4.18 (dd, *J* = 10.8 Hz, 6.8 Hz, 1H), 3.74 (s, 6H), 3.02 (d, *J* = 6.8 Hz, 1H), 2.55 (m, 1H), 2.25 (s, 3H), 1.83 (s, 3H); ^{13}C NMR (100 MHz, $CDCl_3$): δ 208.2, 148.5, 145.8, 136.0, 132.7, 111.9, 111.5, 89.9, 76.4, 75.0, 58.4, 56.0, 55.9, 31.5, 14.0; HRMS (ESI) calcd. for $C_{15}H_{19}O_5$ $[M+H]^+$: 279.1232; found: 279.1228.

1-(3-Hydroxy-5,6-bis(methoxymethyl)-7-oxabicyclo[2.2.1]heptan-2-yl)ethan-1-one, 20 (Scheme 4): Yield = 82% (40 mg, 0.163 mmol); White solid; mp: 72-74 ˚C; R_f = 0.13 (EtOAc, 100%); IR (ν, cm^{-1}): 3054, 2987, 1714, 1422, 1265, 896, 746; ^{1}H NMR (400MHz, $CDCl_3$): δ 4.68 (d, *J* = 0.9 Hz, 1H), 4.29 (dd, J_1 = 9.6 Hz, J_2 = 7 Hz, 1H), 4.19 (d, *J* = 0.9 Hz, 1H), 3.36-3.19 (m, 10H), 2.94 (d, *J* = 6.9 Hz, 1H), 2.38 (d, *J* = 10.1 Hz, 1H), 2.19 (s, 3H), 2.0-1.94 (m, 2H); ^{13}C NMR (100 MHz, $CDCl_3$): δ 206.2, 84.9, 79.1, 75.6, 70.4, 60.7, 58.9, 58.8, 44.8, 40.5, 31.3; HRMS (ESI) calcd. for $C_{11}H_{20}O_5$ $[M.-H]^-$: 243.1232; found: 243.1237.

6-Acetyl-5-hydroxy-4-methyl-2-phenylhexahydro-1*H*-4,7-epoxyisoindole-1,3(2*H*)-dione, 21a (Scheme 4): Yield = 80% (40 mg, 0.126 mmol); Off-white solid; mp: 252-253 ˚C; R_f = 0.4 (EtOAc, 100%); IR (ν, cm^{-1}): 3054, 2987, 1713, 1422, 1265, 896, 746; ^1H NMR (300MHz, DMSO-d_6): δ 7.50-7.38 (m, 3H), 7.19 (d, *J* = 7.2 Hz, 2H), 5.64 (d, *J* = 6.2 Hz, 1H), 4.82 (s, 1H), 4.26-4.22 (m, 1H), 3.23-3.16 (m, 2H), 2.97 (d, *J* = 6.9 Hz, 1H), 2.07 (s, 3H), 1.35 (s, 3H); ^{13}C NMR (75 MHz, DMSO-d_6): δ 204.2, 176.3, 175.0, 132.2, 128.9, 128.3, 126.8, 88.0, 77.0, 75.2, 60.1, 50.7, 48.5, 30.4, 12.7; HRMS (ESI) calcd. for $C_{17}H_{18}NO_5$ $[M+H]^+$: 316.1179; Found: 316.1185.

6-Acetyl-4-ethyl-5-hydroxy-2-phenylhexahydro-1*H*-4,7-epoxyisoindole-1,3(2*H*)-dione, 21b (Scheme 4): Yield = 70% (35 mg, 0.106 mmol); Off-white solid; mp: 238-240 ˚C; R_f = 0.4 (EtOAc, 100%); IR (ν, cm^{-1}): 3054, 2987, 1716, 1422, 1265, 896; ^1H NMR (300MHz, DMSO-d_6): δ 7.55-7.35 (m, 3H), 7.25-7.10 (m, 2H), 5.61 (d, *J* = 5.3 Hz, 1H), 4.83(s, 1H), 4.5-4.3 (m, 1H), 3.25-3.05 (m, 3H), 2.2-1.95 (m, 4H), 1.65-1.45 (m, 1H), 1.15-0.95(bs, 3H); ^{13}C NMR (75 MHz, DMSO-d_6): δ 204.2, 176.2, 174.9, 132.2, 128.9, 128.3, 126.8, 91.7, 76.9, 73.2, 59.9, 50.4, 47.0, 30.4, 20.1, 8.3; HRMS (EI) calcd. for $C_{18}H_{19}NO_5$ $[M]^+$: 329.1263; Found: 329.1277.

1-(2-Acetyl-3-hydroxy-1,2,3,4-tetrahydro-1,4-epiminonaphthalen-9-yl)-2,2-dimethylpropan-1-one, 22 (Scheme 4): Yield = 66% (40 mg, 0.139 mmol); Light brown solid; mp: 162-164 ˚C; R_f = 0.34 (EtOAc-hexanes, 1:1); IR (ν, cm^{-1}): 3054, 2987, 1715, 1637, 1422, 1265, 896, 738; ^1H NMR (400MHz, $CDCl_3$): δ 7.32-7.30 (m, 1H), 7.22-7.14 (m, 3H), 5.69 (s, 1H), 5.42 (s, 1H), 4.33 (dd, J_1 = 9 Hz, J_2 = 7.1 Hz, 1H), 3.4 (br s, 1H); 2.85 (d, *J* = 6.9 Hz, 1H), 2.26 (s, 3H), 1.2 (s, 9H); ^{13}C NMR (100 MHz, $CDCl_3$): δ 207 (C=O, from HMBC), 179 (N-C=O, from HMBC) 145.5, 141.6, 127.6, 127.0, 121.6, 119.4, 78.0 (C-H from HMBC), 74.0 (C-H, from

HSQC), 68.7, 61.8, 39.5, 31.1, 27.7; HRMS (ESI) calcd. for $C_{17}H_{22}NO_3$ $[M+H]^+$: 288.1599; found: 2888.1610
