# Supplementary material for: N–O Cleavage reactions of heterobicycloalkene-fused 2-isoxazolines
Source: Beilstein J Org Chem. 2014 Sep 16;10:2200–5. doi: 10.3762/bjoc.10.227 (PMC4168885; doi:10.3762/bjoc.10.227)
Supplement: File 2 — NMR Spectra. [file Beilstein_J_Org_Chem-10-2200-s002.pdf]

Supporting Information

for

N–O Cleavage reactions of heterobicycloalkene-fused 2-isoxazolines

Jaipal R. Nagireddy, Geoffrey K. Tranmer, Emily Carlson and William Tam*

Address: Guelph-Waterloo Centre for Graduate Work in Chemistry and Biochemistry, Department of Chemistry and Biochemistry, University of Guelph, Guelph, Ontario, N1G 2W1, Canada

Email: William Tam - wtam@uoguelph.ca

*Corresponding author

NMR Spectra

Table of Contents

^{1}H and ^{13}C NMR Spectra of Compounds **16a-k** S2-S12

^{1}H and ^{13}C NMR Spectra of Compound **20** S13

^{1}H and ^{13}C NMR Spectra of Compounds **21a-b** S14-S15

^{1}H, ^{13}C, HMBC and HSQC Spectra of Compound **22** S16-S17

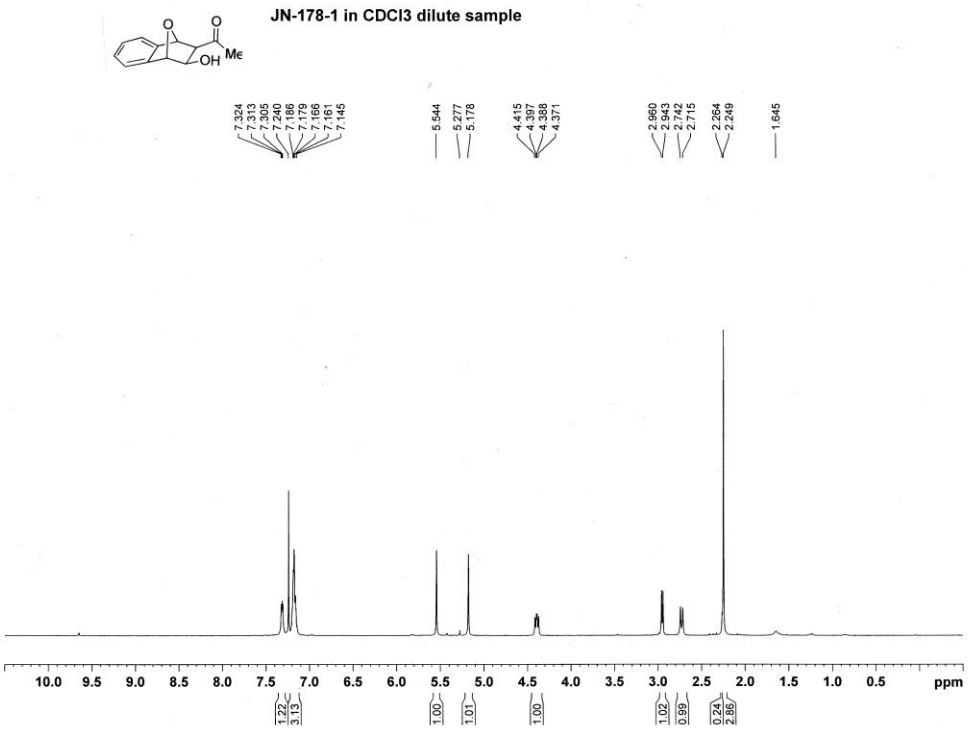

400 MHz ^{1}H NMR spectrum of **16a** in $CDCl_3$

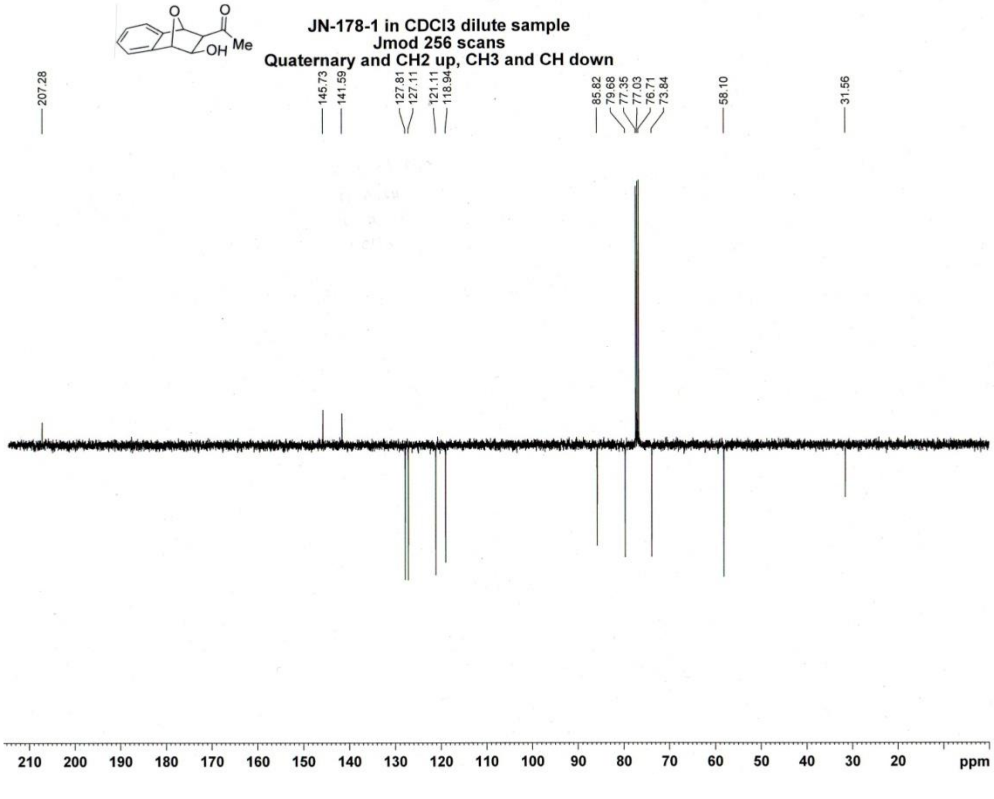

100 MHz ^{13}C NMR spectrum of **16a** in $CDCl_3$

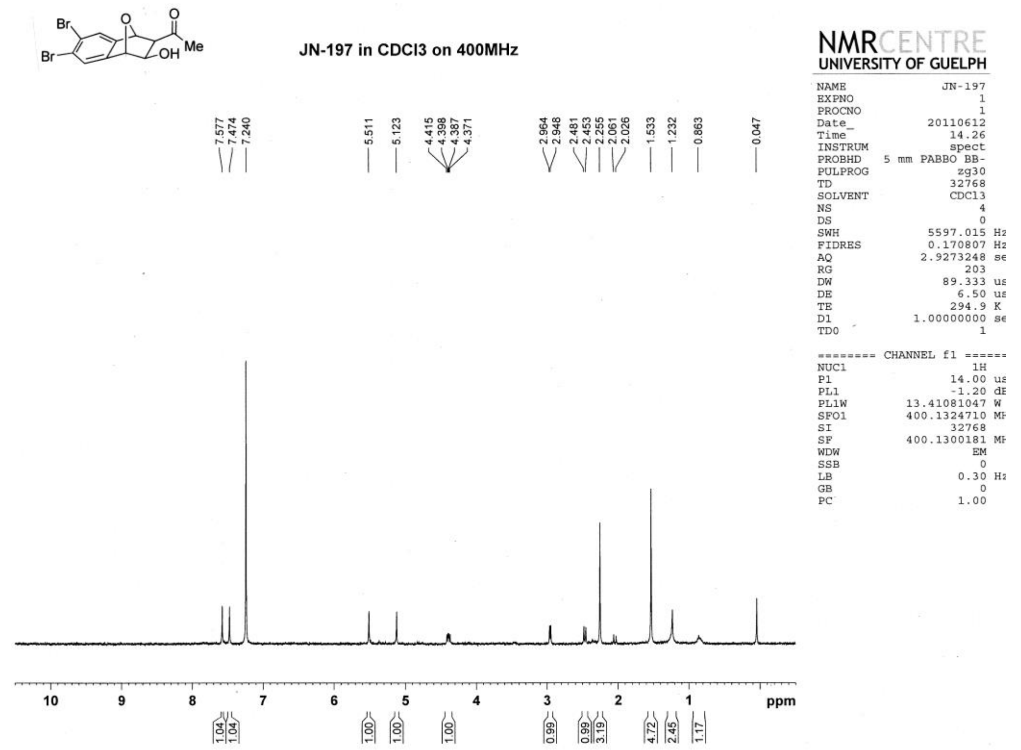

400 MHz ^{1}H NMR spectrum of **16b** in $CDCl_3$

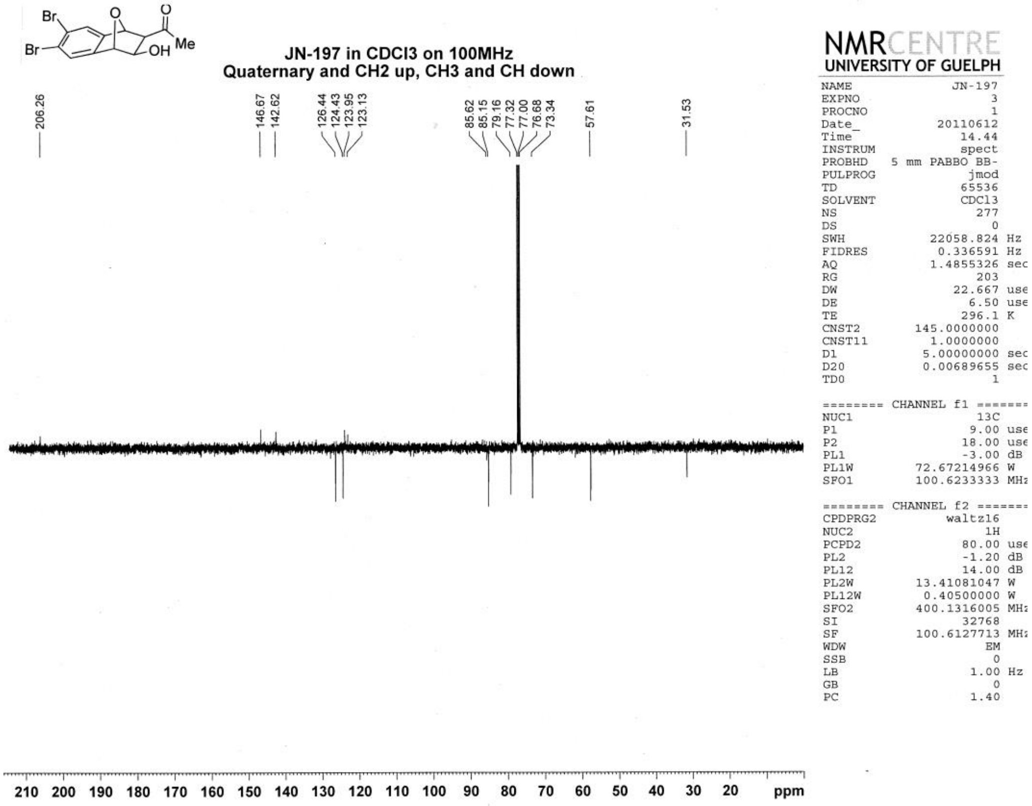

100 MHz ^{13}C NMR spectrum of **16b** in $CDCl_3$

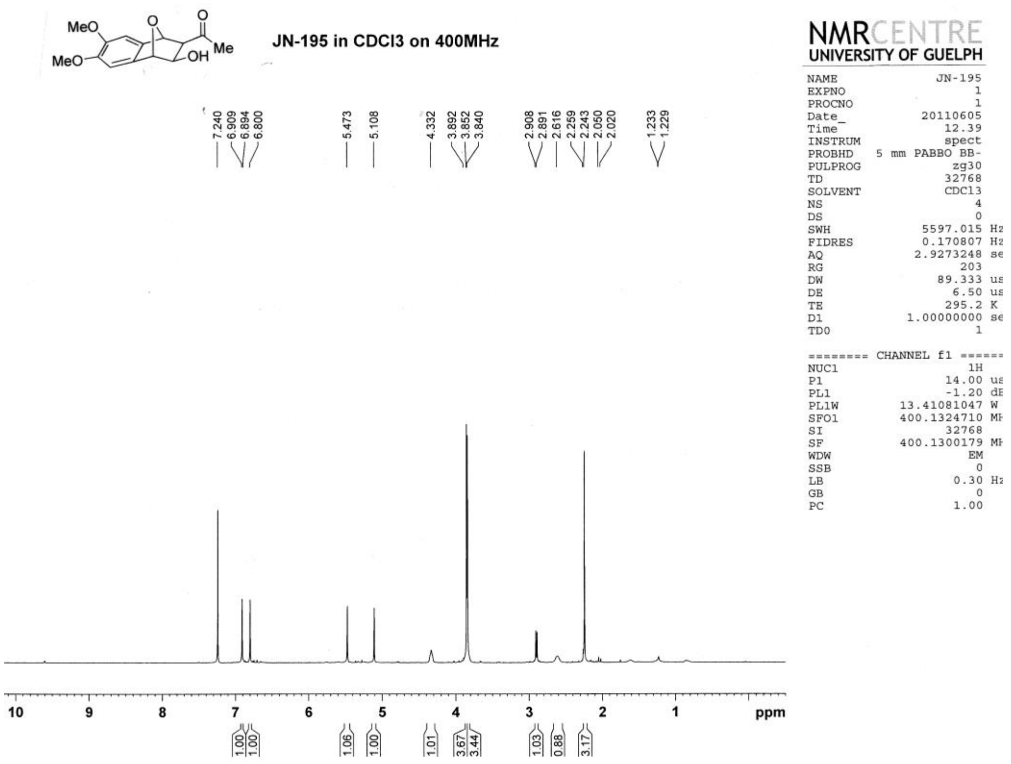

400 MHz ^{1}H NMR spectrum of **16c** in $CDCl_3$

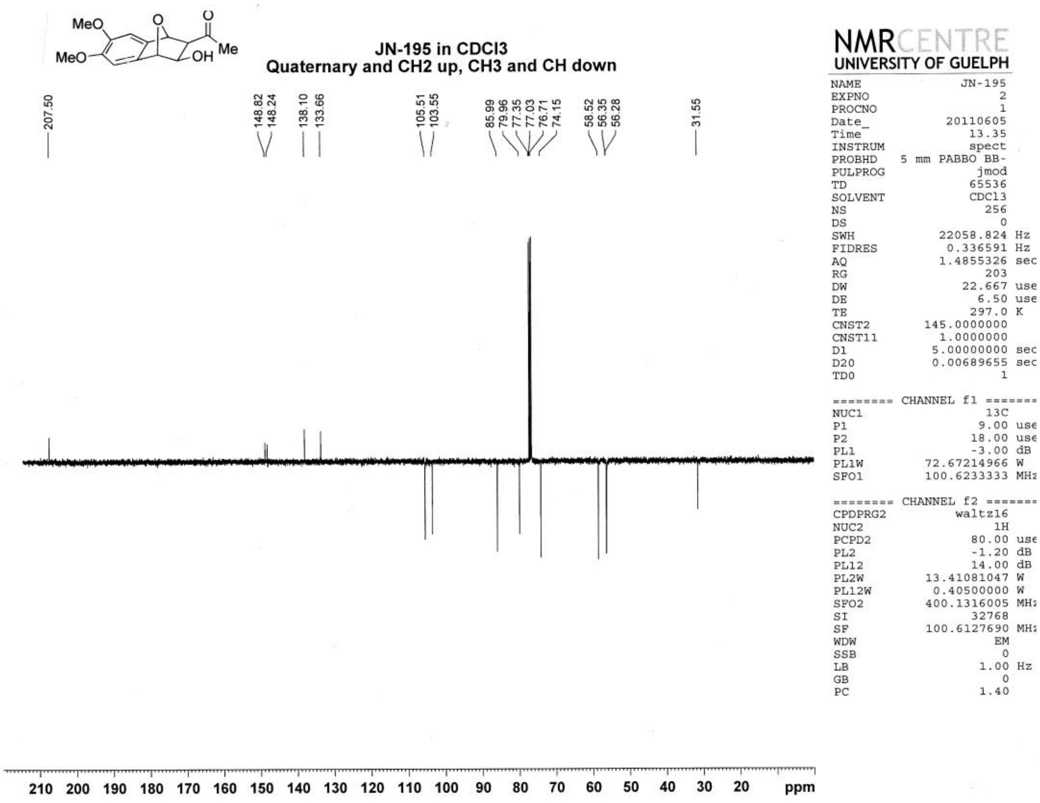

100 MHz ^{13}C NMR spectrum of **16c** in $CDCl_3$

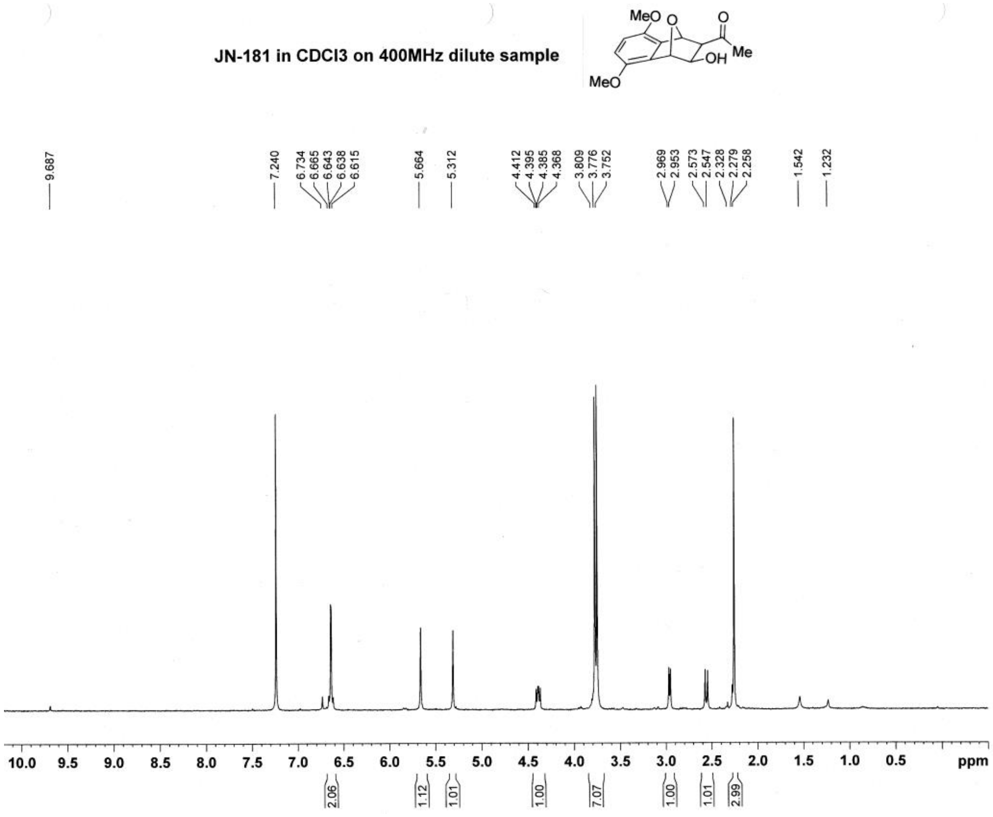

400 MHz ^{1}H NMR spectrum of **16d** in $CDCl_3$

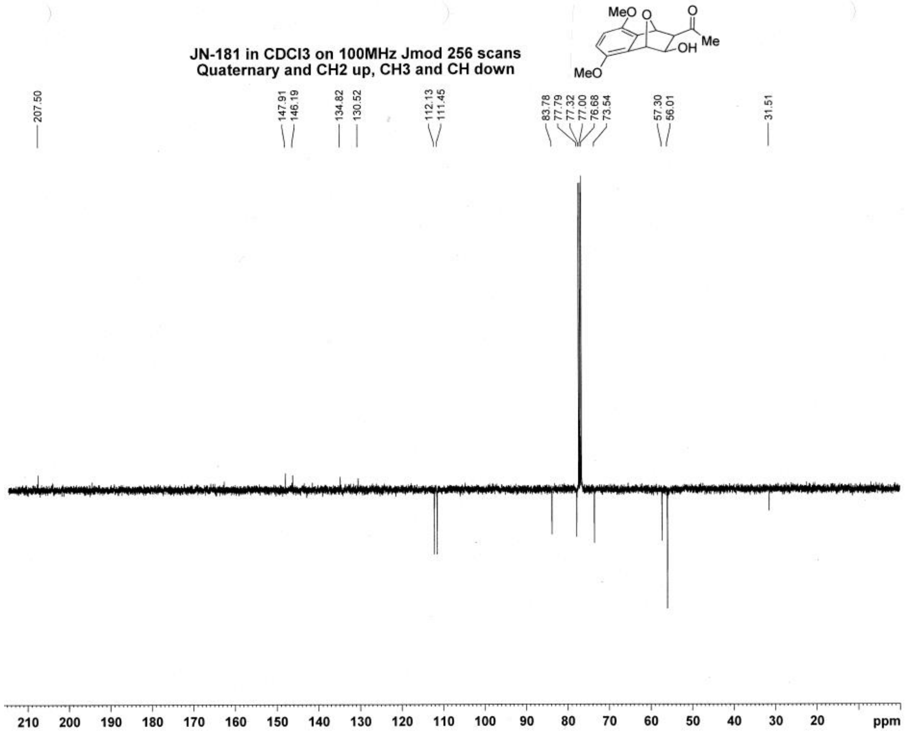

100 MHz ^{13}C NMR spectrum of **16d** in $CDCl_3$

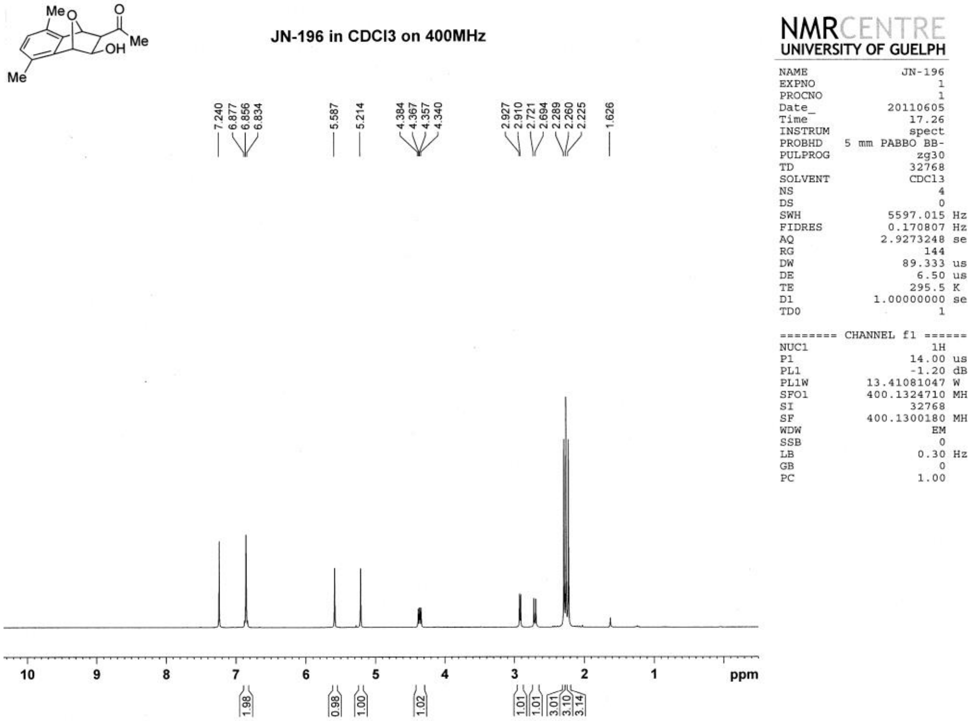

400 MHz ^{1}H NMR spectrum of **16e** in $CDCl_3$

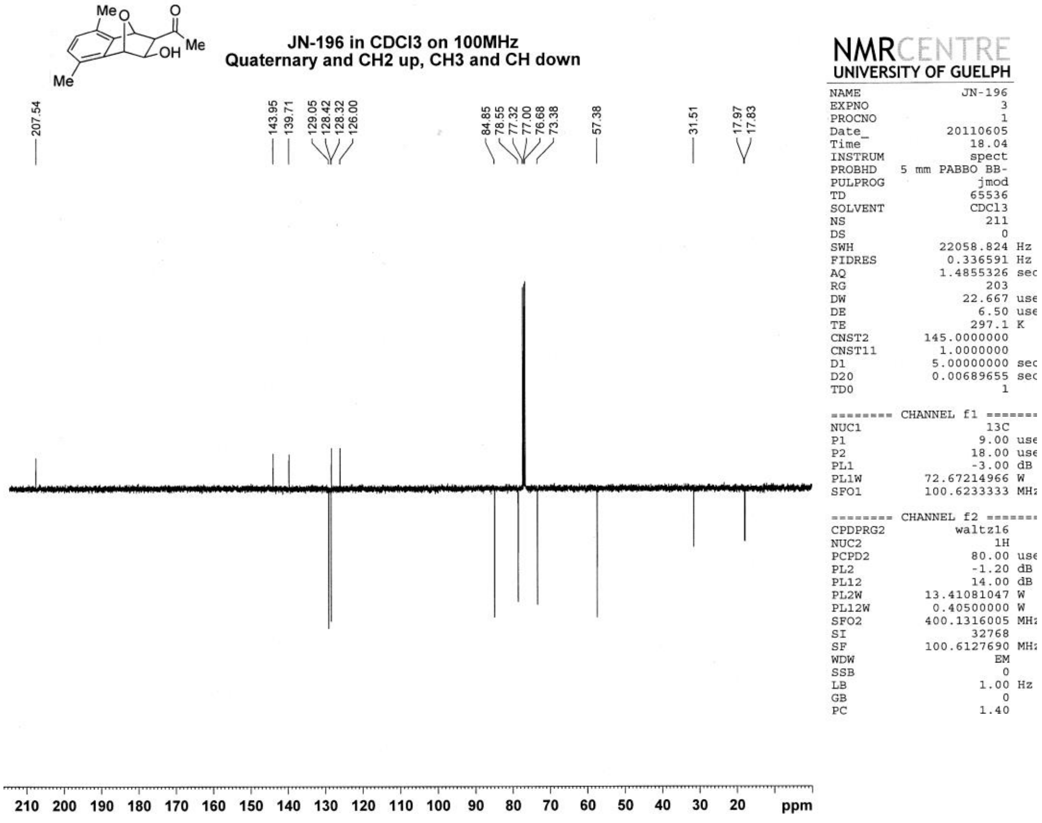

100 MHz ^{13}C NMR spectrum of **16e** in $CDCl_3$

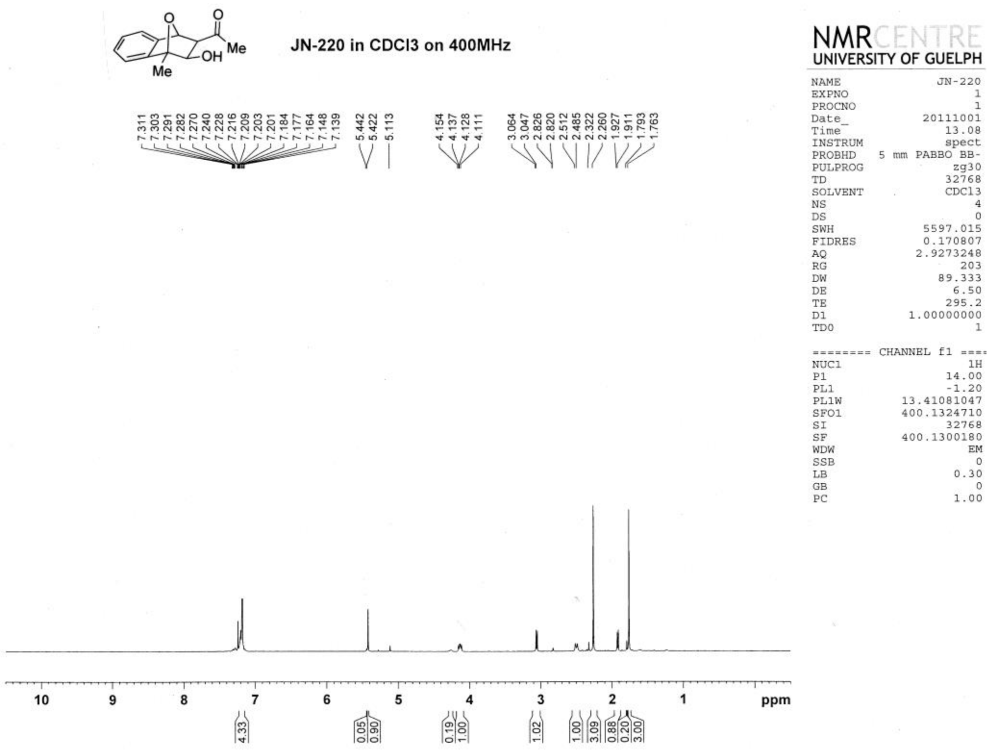

400 MHz ^{1}H NMR spectrum of **16f** in $CDCl_3$

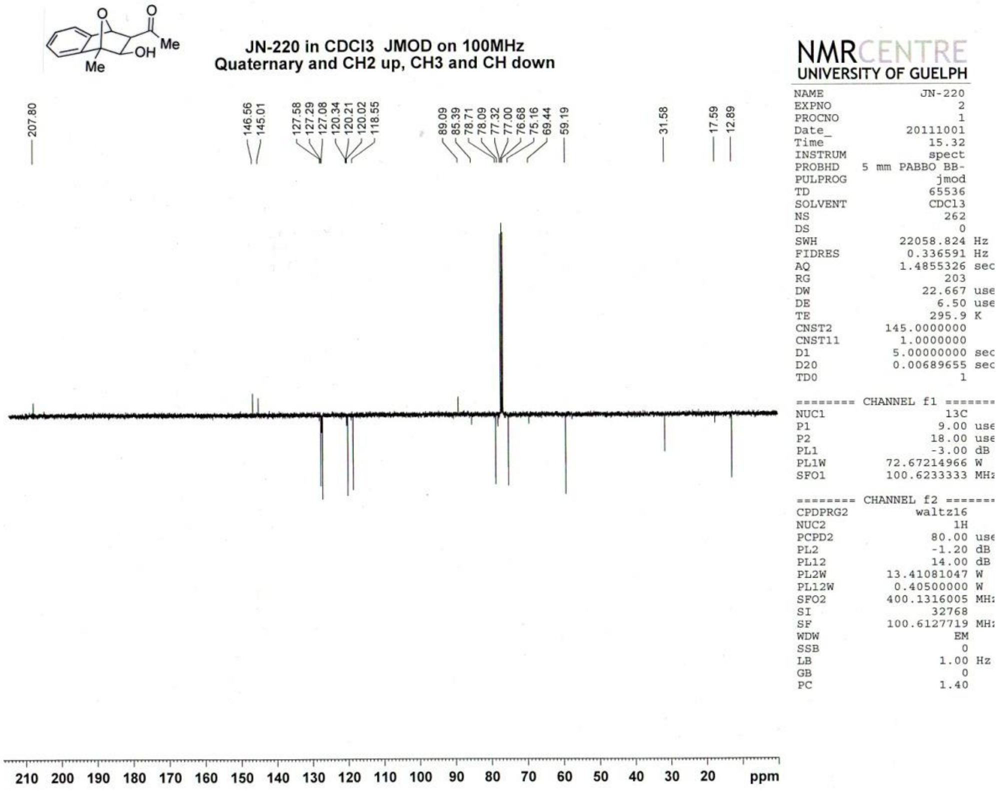

100 MHz ^{13}C NMR spectrum of **16f** in $CDCl_3$

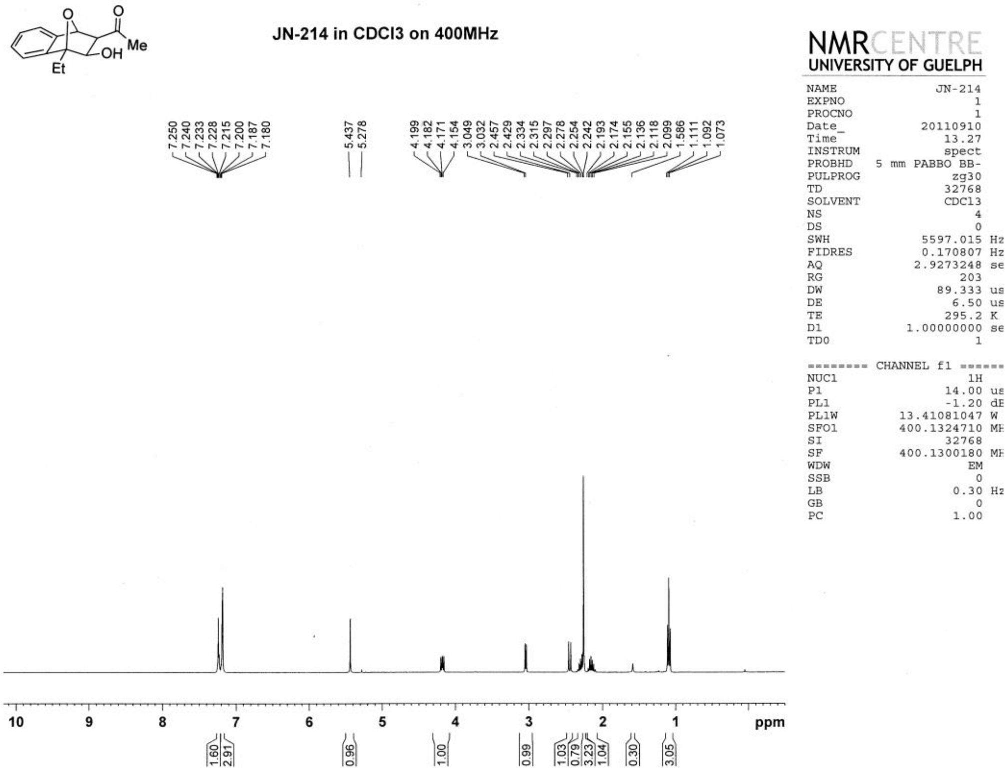

400 MHz ^{1}H NMR spectrum of **16g** in $CDCl_3$

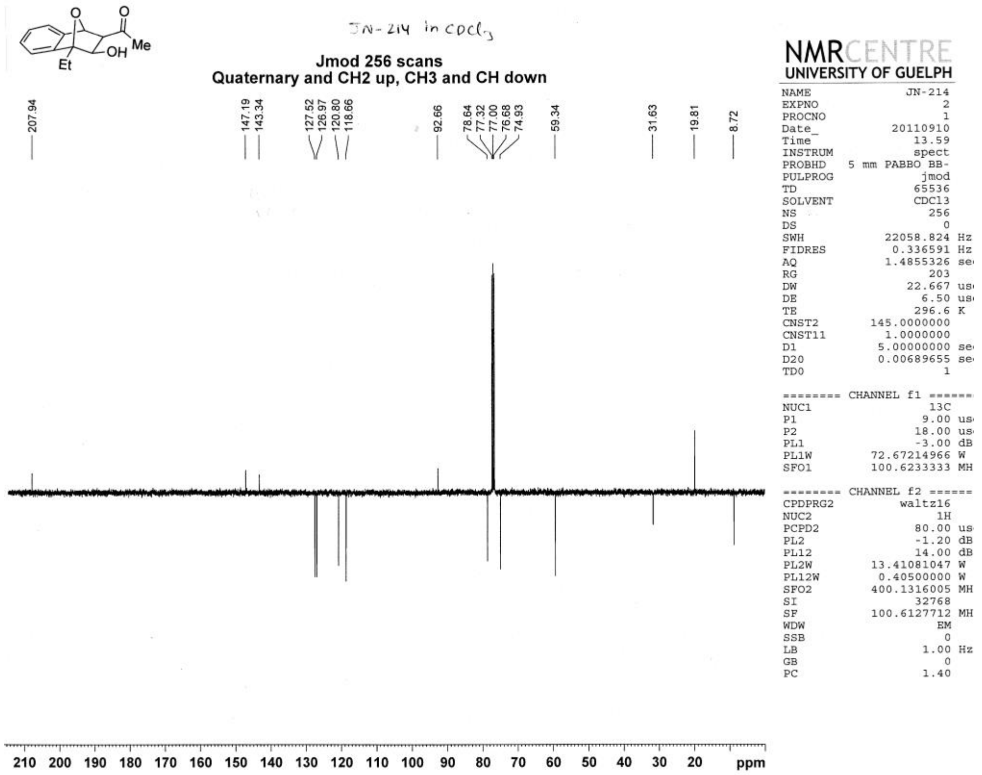

100 MHz ^{13}C NMR spectrum of **16g** in $CDCl_3$

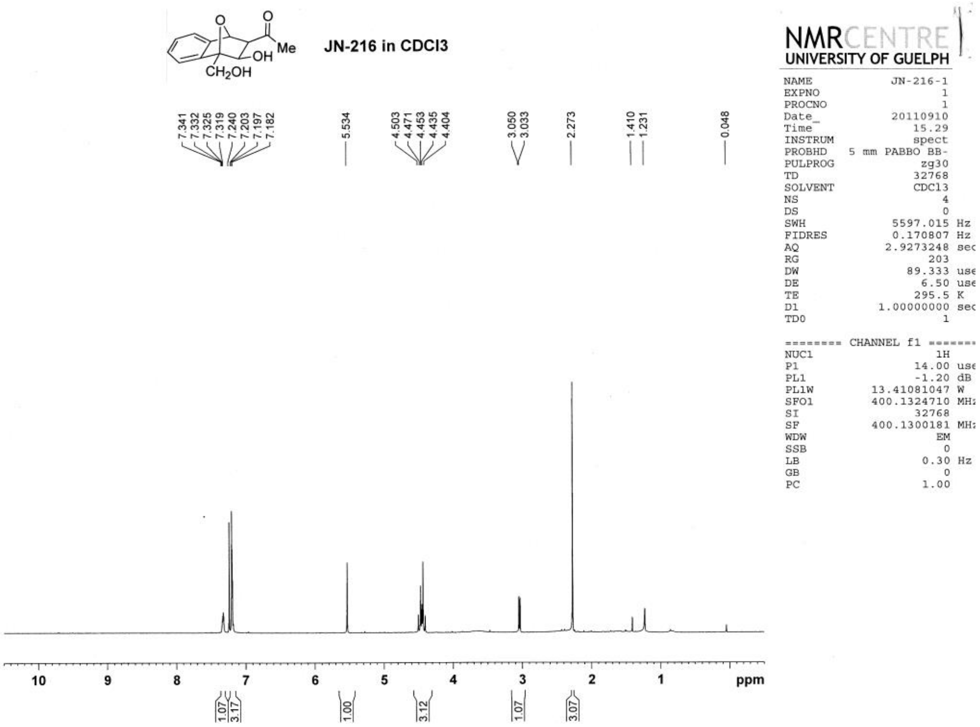

400 MHz ^{1}H NMR spectrum of **16h** in $CDCl_3$

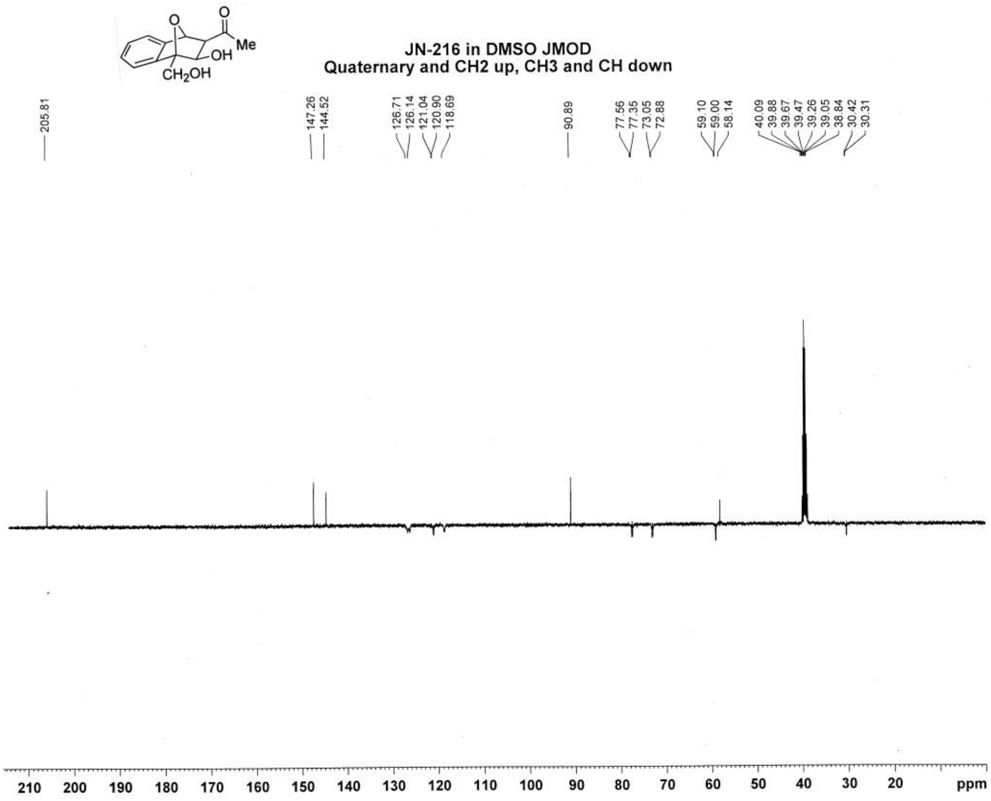

100 MHz ^{13}C NMR spectrum of **16h** in DMSO-d_6

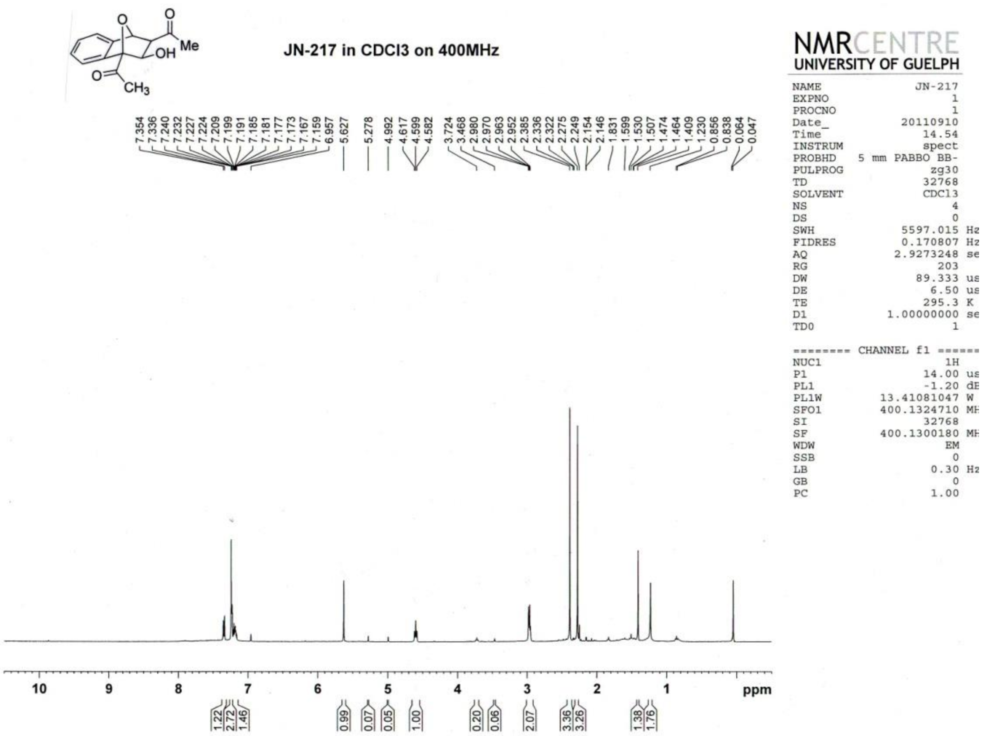

400 MHz ^{1}H NMR spectrum of **16i** in $CDCl_3$

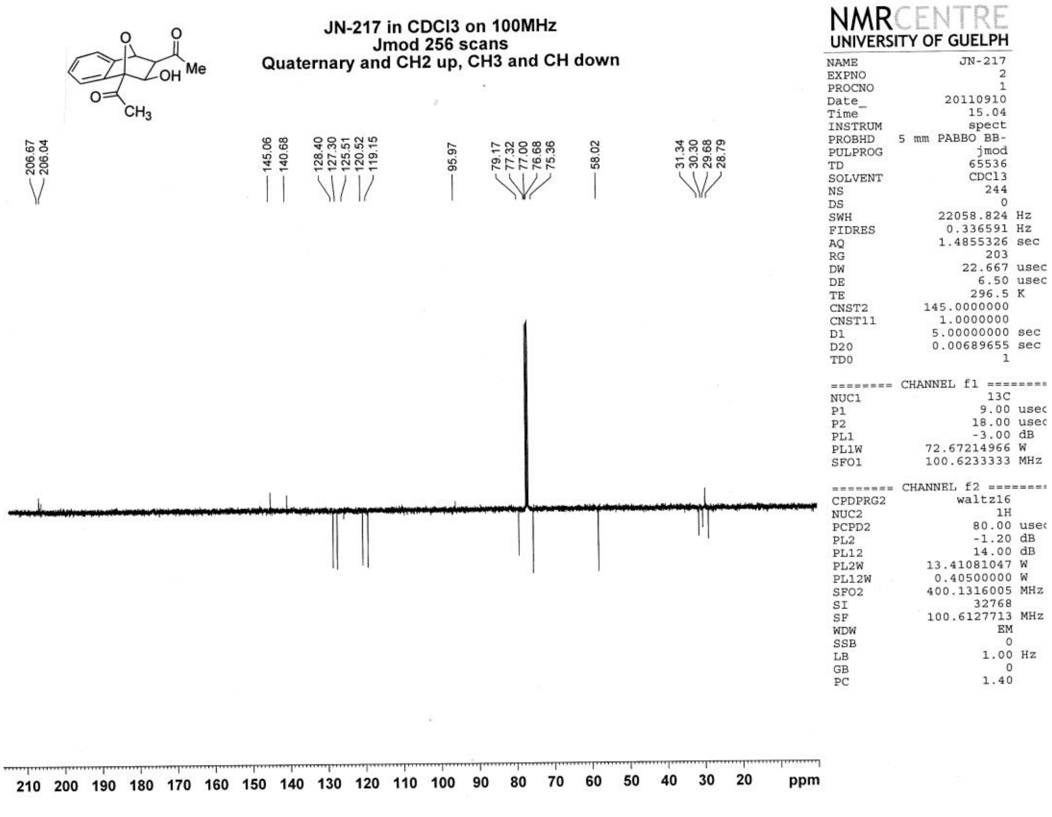

100 MHz ^{13}C NMR spectrum of **16i** in $CDCl_3$

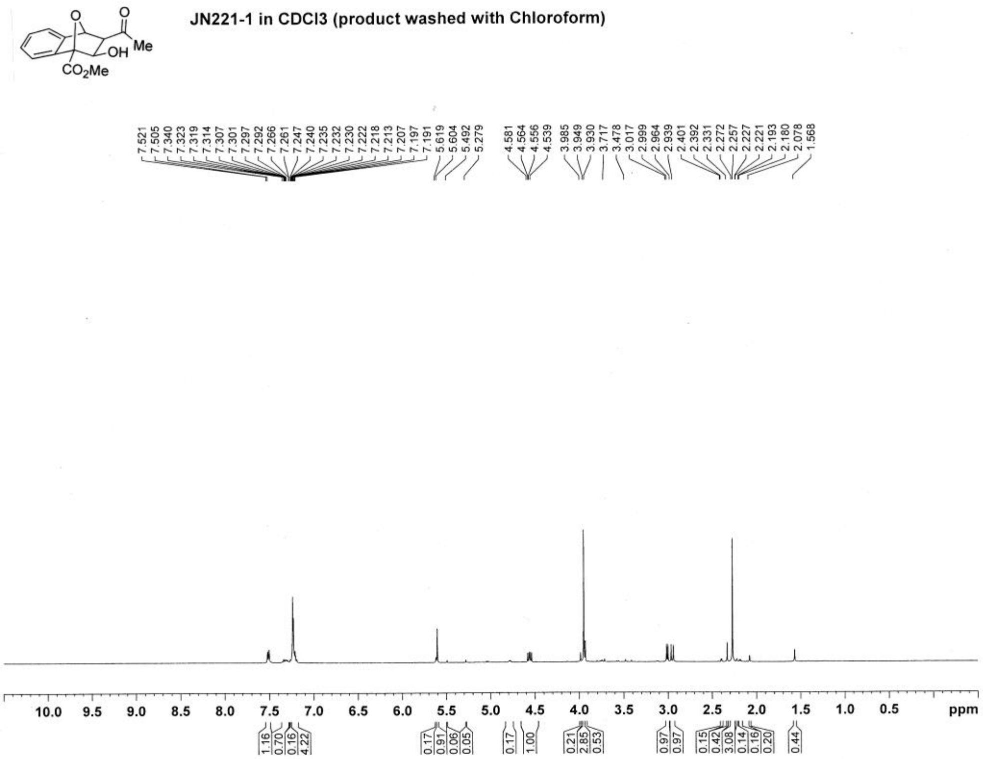

400 MHz ^{1}H NMR spectrum of **16j** in $CDCl_3$

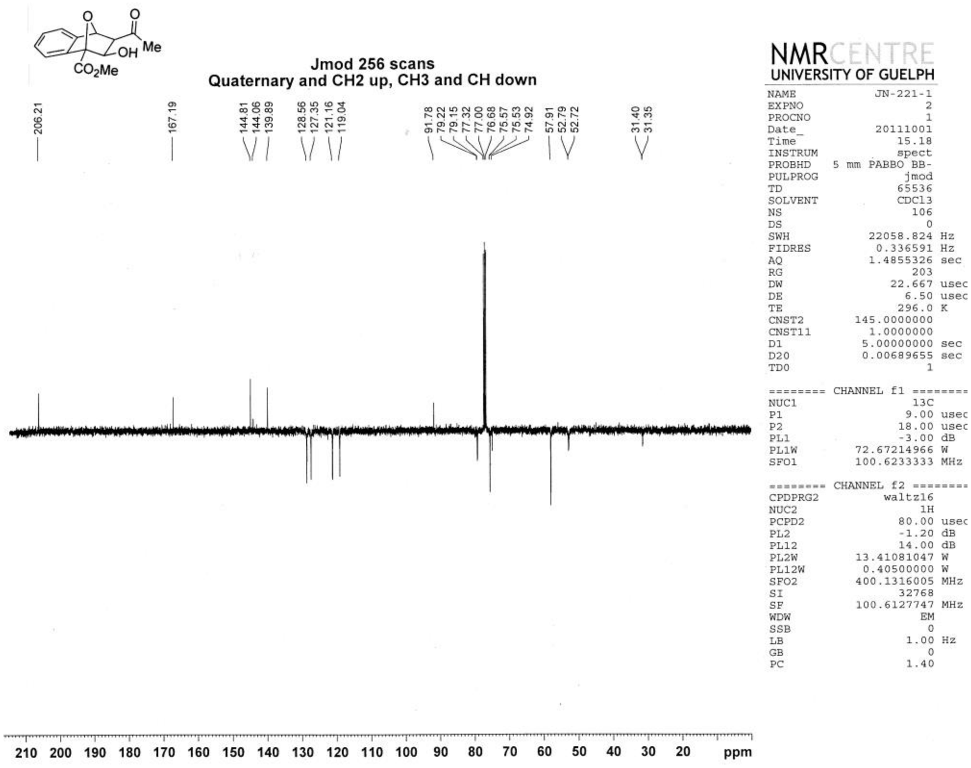

100 MHz ^{13}C NMR spectrum of **16j** in $CDCl_3$

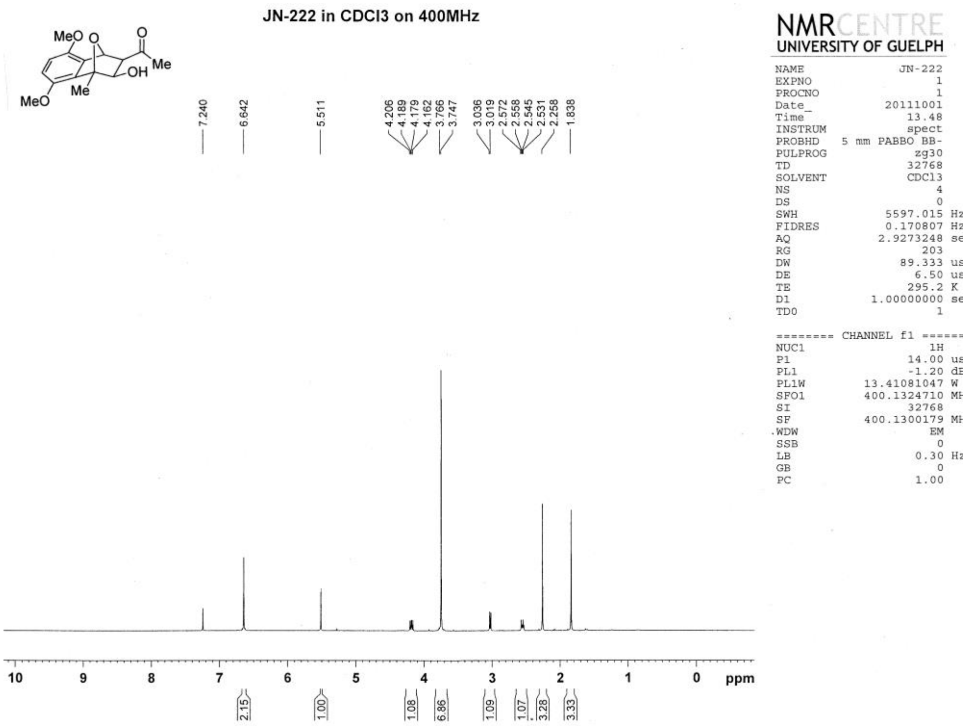

400 MHz ^{1}H NMR spectrum of **16k** in $CDCl_3$

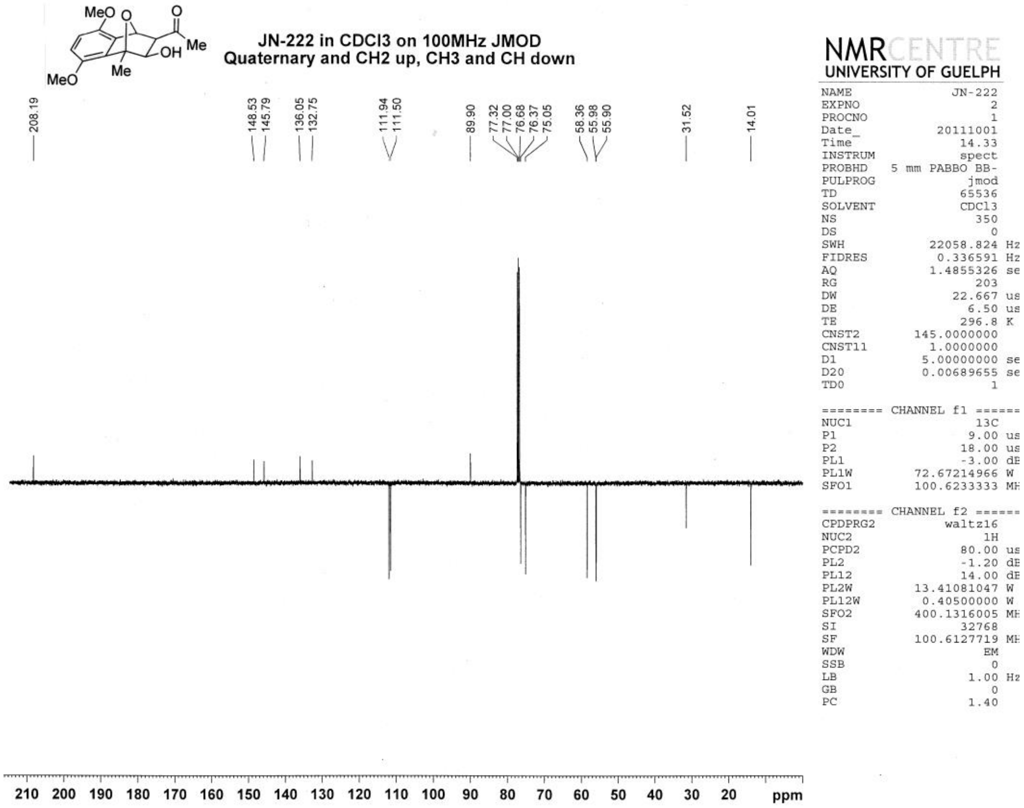

100 MHz ^{13}C NMR spectrum of **16k** in $CDCl_3$

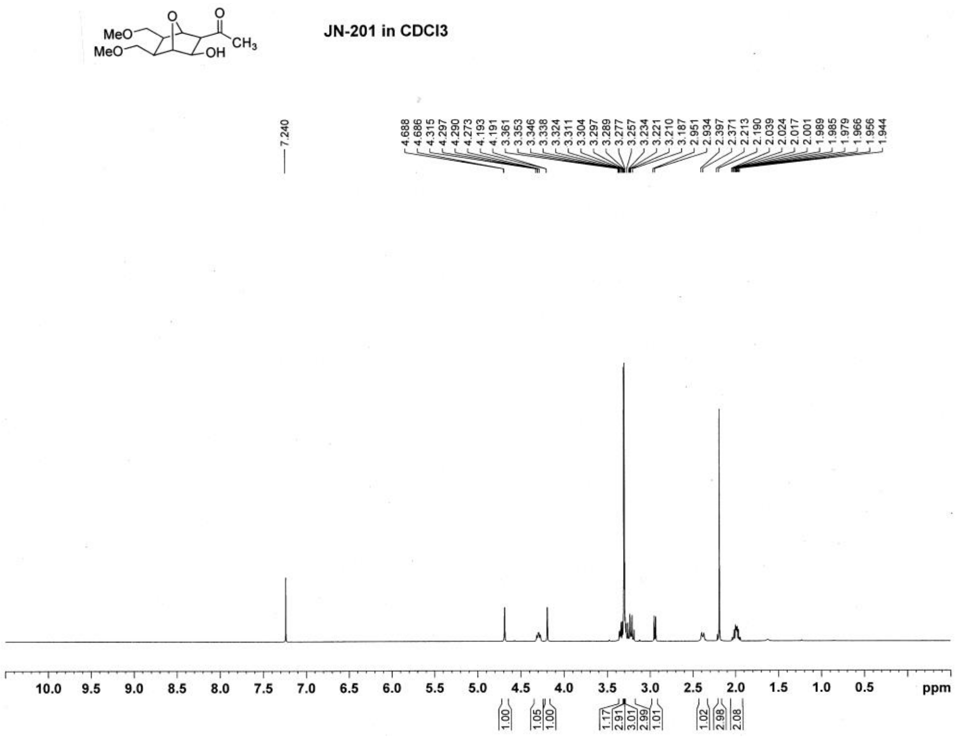

400 MHz ^{1}H NMR spectrum of **20** in $CDCl_3$

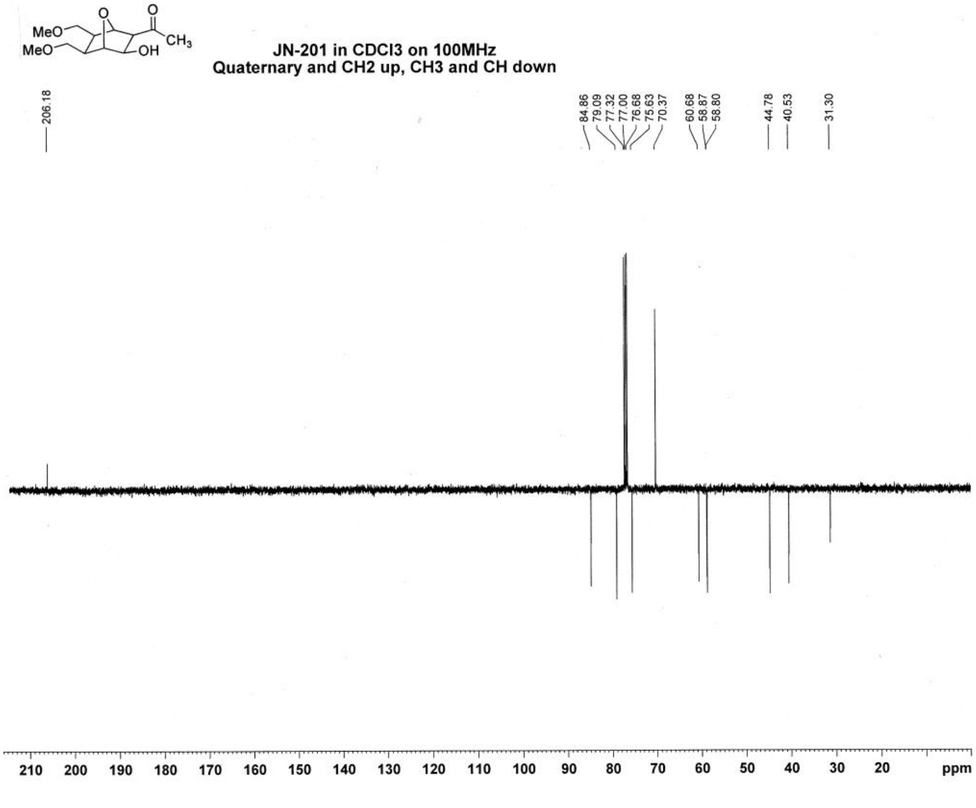

100 MHz ^{13}C NMR spectrum of **20** in $CDCl_3$

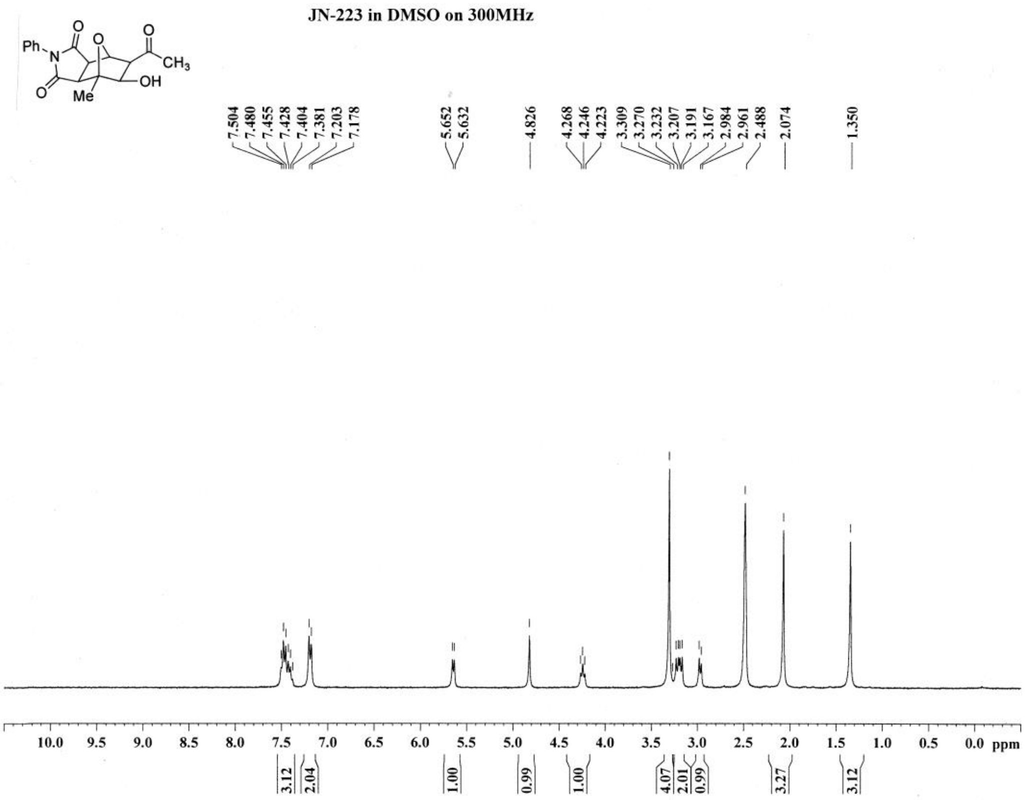

300 MHz ^{1}H NMR spectrum of **21a** in DMSO-d_6

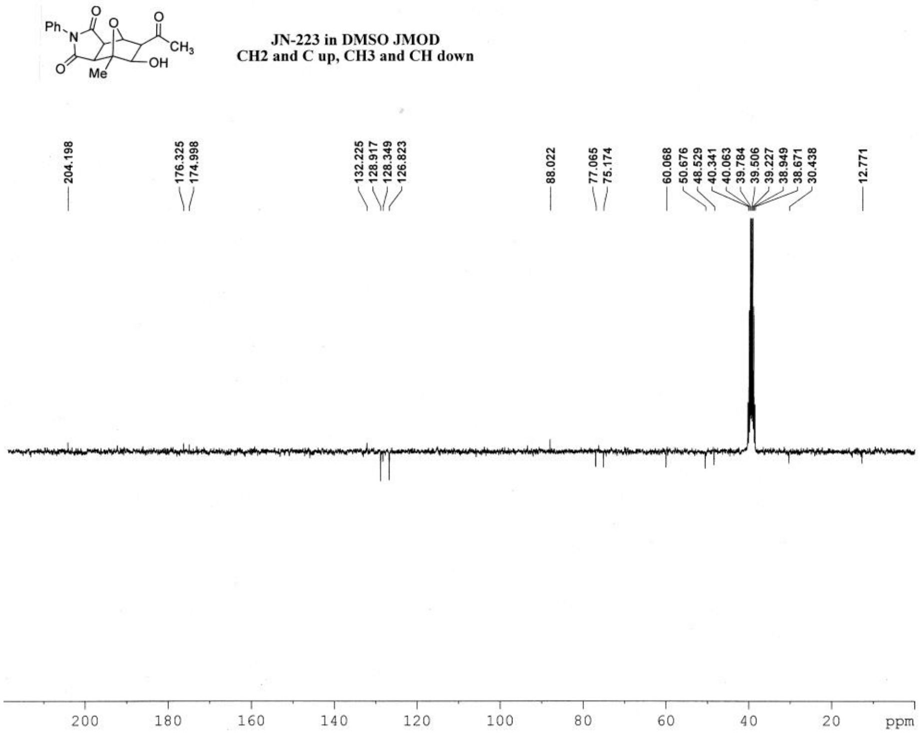

75 MHz ^{13}C NMR spectrum of **21a** in DMSO-d_6

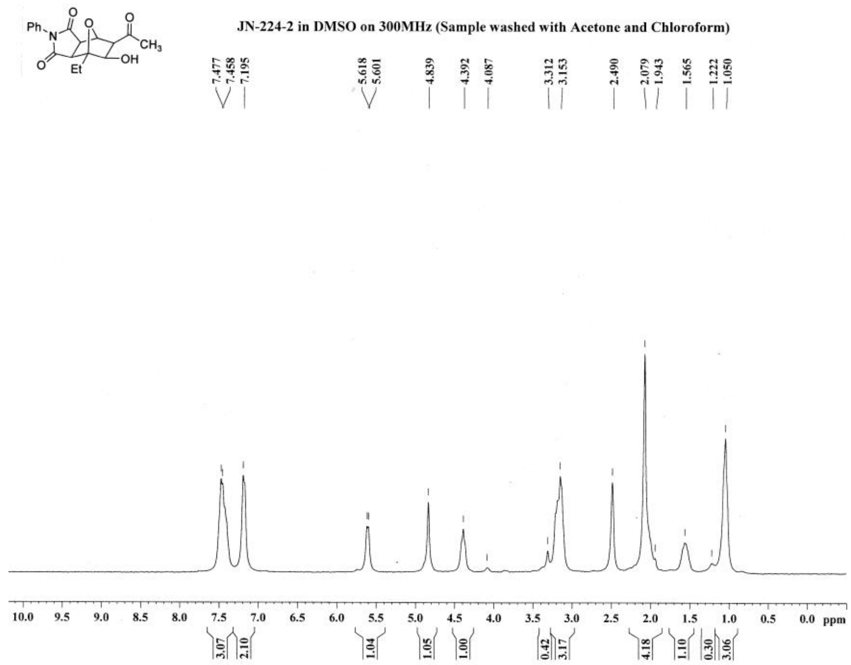

300 MHz ^{1}H NMR spectrum of **21b** in DMSO-d_6

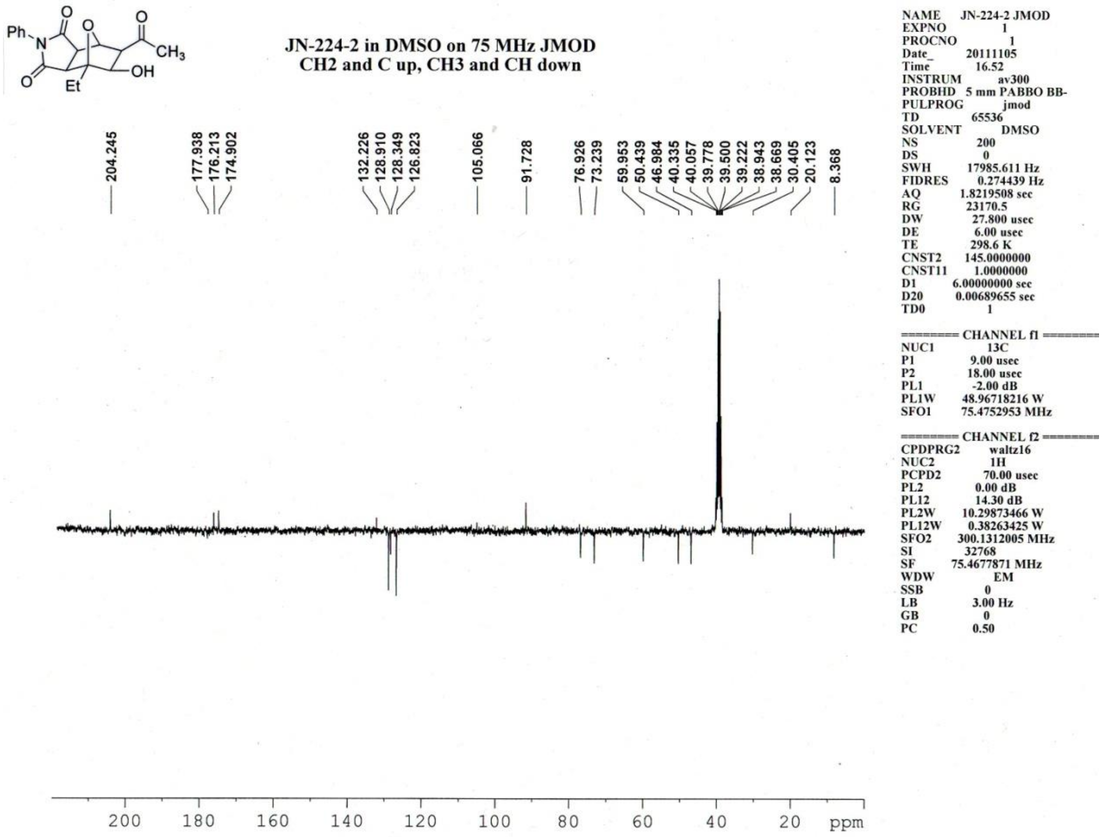

75 MHz ^{13}C NMR spectrum of **21b** in DMSO-d_6

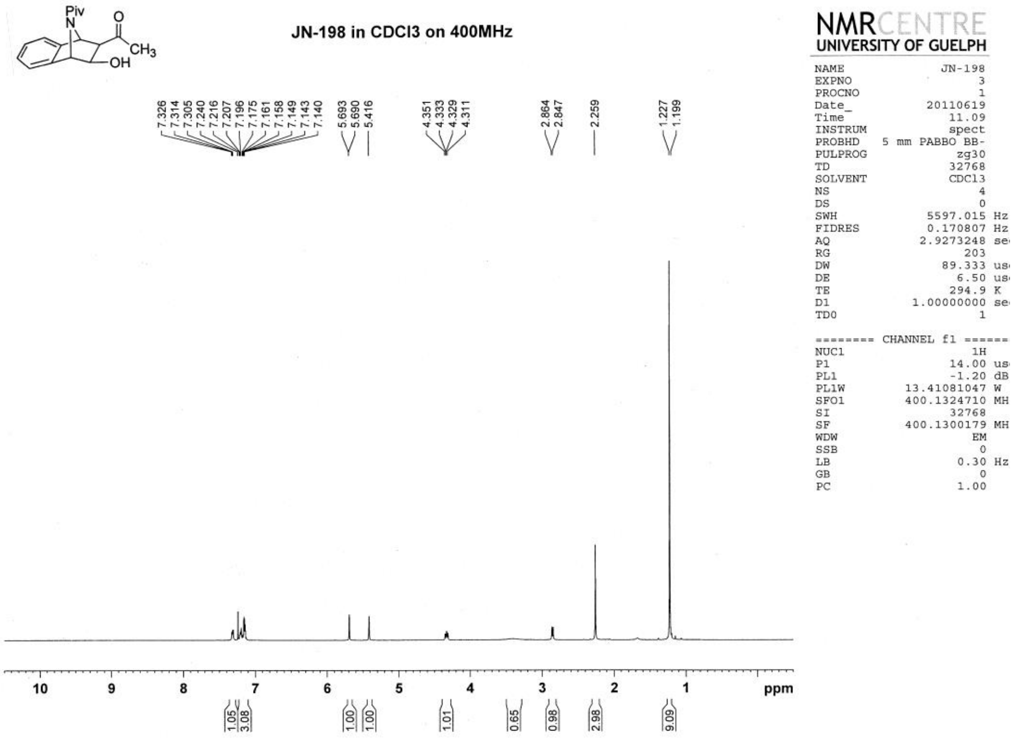

400 MHz ^{1}H NMR spectrum of **22** in $CDCl_3$

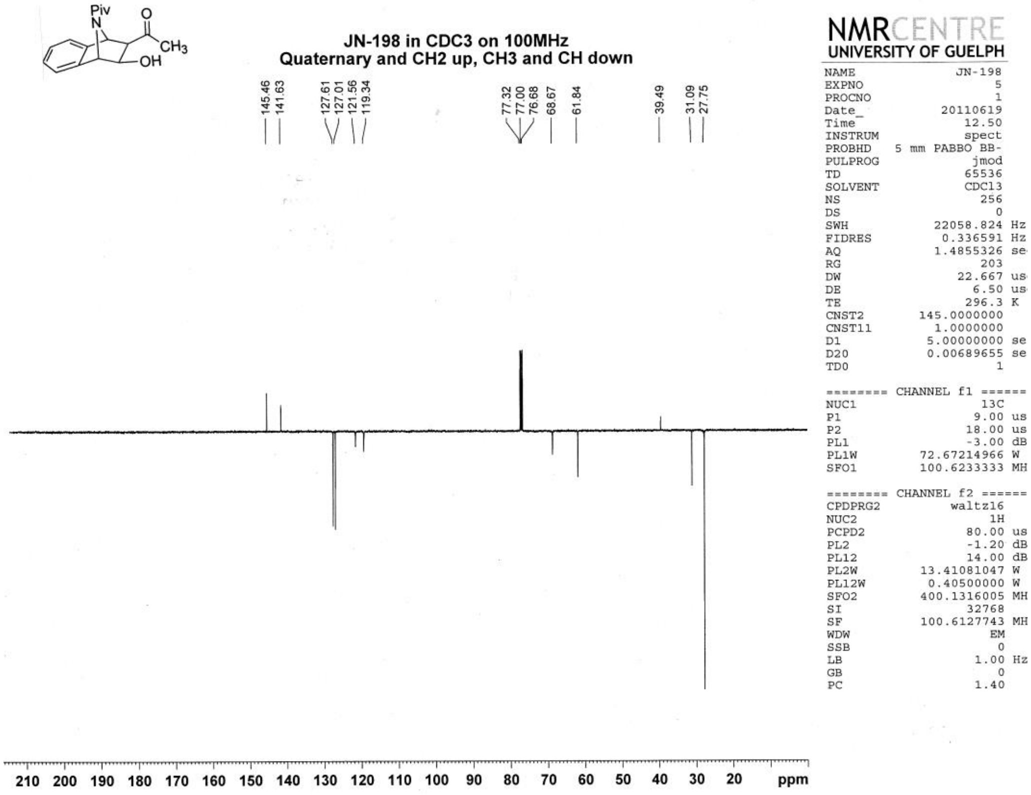

100 MHz ^{13}C NMR spectrum of **22** in $CDCl_3$

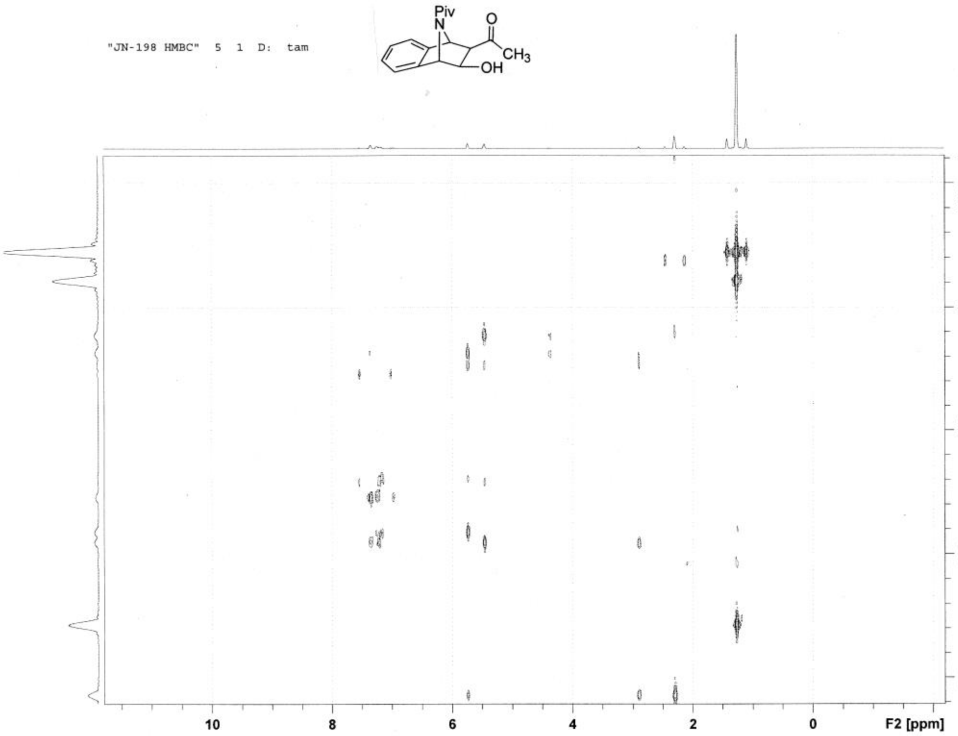

400 MHz HMBC spectrum of **22** in $CDCl_3$

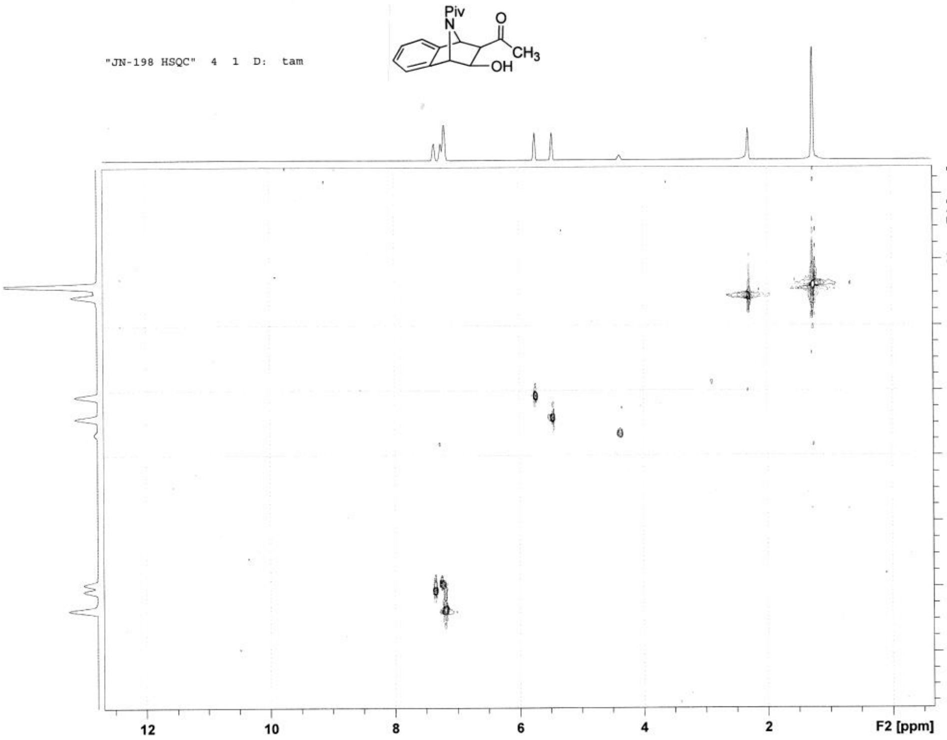

400 MHz HSQC spectrum of **22** in $CDCl_3$
